# Diverse Evolutionary Trajectories for Small RNA Biogenesis Genes in the Oomycete Genus *Phytophthora*

**DOI:** 10.3389/fpls.2016.00284

**Published:** 2016-03-15

**Authors:** Stephanie R. Bollmann, Yufeng Fang, Caroline M. Press, Brett M. Tyler, Niklaus J. Grünwald

**Affiliations:** ^1^Horticultural Crop Research Unit, USDA-Agricultural Research ServiceCorvallis, OR, USA; ^2^Department of Botany and Plant Pathology and Center for Genome Biology and Biocomputing, Oregon State UniversityCorvallis, OR, USA; ^3^Interdisciplinary Ph.D. Program in Genetics, Bioinformatics and Computational Biology, Virginia TechBlacksburg, VA, USA

**Keywords:** dicer, RDR, evolution, *Phytophthora*, small RNA, stramenopile

## Abstract

Gene regulation by small RNA pathways is ubiquitous among eukaryotes, but little is known about small RNA pathways in the Stramenopile kingdom. *Phytophthora*, a genus of filamentous oomycetes, contains many devastating plant pathogens, causing multibillion-dollar damage to crops, ornamental plants, and natural environments. The genomes of several oomycetes including *Phytophthora* species such as the soybean pathogen *P. sojae*, have been sequenced, allowing evolutionary analysis of small RNA-processing enzymes. This study examined the evolutionary origins of the oomycete small RNA-related genes Dicer-like (*DCL)*, and RNA-dependent RNA polymerase (*RDR)* through broad phylogenetic analyses of the key domains. Two Dicer gene homologs, *DCL1* and *DCL2*, and one RDR homolog were cloned and analyzed from *P. sojae*. Gene expression analysis revealed only minor changes in transcript levels among different life stages. Oomycete DCL1 homologs clustered with animal and plant Dicer homologs in evolutionary trees, whereas oomycete DCL2 homologs clustered basally to the tree along with Drosha homologs. Phylogenetic analysis of the RDR homologs confirmed a previous study that suggested the last common eukaryote ancestor possessed three RDR homologs, which were selectively retained or lost in later lineages. Our analysis clarifies the position of some Unikont and Chromalveolate RDR lineages within the tree, including oomycete homologs. Finally, we analyzed alterations in the domain structure of oomycete Dicer and RDR homologs, specifically focusing on the proposed domain transfer of the DEAD-box helicase domain from Dicer to RDR. Implications of the oomycete domain structure are discussed, and possible roles of the two oomycete Dicer homologs are proposed.

## Introduction

Gene regulation through the action of small RNAs is ubiquitous among eukaryotes and is well characterized in plants, animals, and fungi but not in other eukaryotic lineages (Ghildiyal and Zamore, 2009). The three most prevalent types of small RNAs are short interfering RNAs (siRNA), microRNAs (miRNA), and PIWI-interacting RNAs (piRNA), with the siRNA pathway being common to all investigated eukaryotic kingdoms. SiRNAs act in silencing of repetitive sequences such as transposons and in antiviral defense (Hamilton and Baulcombe, 1999). miRNAs regulate many biological processes, with defects in miRNAs causing severe phenotypes such as autoimmune disease and cancer (Alemdehy et al., 2015; Mehta et al., 2015). miRNA expression is often specific to a tissue or developmental stage (Lee et al., 1993; Wightman et al., 1993). PiRNAs, which are specific to metazoans, are required for germline stem cell maintenance and promotion of cell division (Aravin et al., 2001; Vagin et al., 2006).

To date, small RNAs have been described for plants, animals, fungi, and some protists, yet little is known about small RNA biology in the Stramenopile kingdom, which includes golden-brown algae, diatoms, brown algae, and oomycetes. Many oomycetes, including those in the genus *Phytophthora*, are devastating plant pathogens, affecting diverse hosts and causing multibillion dollar damage to crops, ornamental plants, and natural environments. A number of oomycetes are also pathogens of aquatic hosts, such as the fish pathogen *Saprolegnia parasitica*. Notable *Phytophthora* species include *Phytophthora infestans* that infects potatoes and tomatoes and caused the Irish potato famine, *P. sojae* that infect soybeans, and *P. ramorum* that causes sudden oak death (Tyler, 2007; Fry, 2008; Grünwald et al., 2008). The genomes of these three *Phytophthora* species have been sequenced (Tyler et al., 2006; Haas et al., 2009), as well as the more distantly related oomycetes *Hyaloperonospora arabidopsidis* (Baxter et al., 2010), *Pythium ultimum* (Lévesque et al., 2010), *Albugo candida* (Links et al., 2011), and *S. parasitica* (Jiang et al., 2013), opening the door to investigation of oomycete small RNA pathways through comparative genomic approaches.

Small RNA biogenesis requires processing by two key enzymes: Dicer [or dicer-like (DCL) in non-metazoans] and RNA-dependent RNA polymerase. Briefly, siRNAs originate from an inverted repeat transcript, sense/anti-sense transcription of the same template, or the action of an RNA-dependent RNA polymerase (RDR) on an ssRNA template (Makeyev and Bamford, 2002; Ghildiyal and Zamore, 2009). All siRNAs are processed by Dicer (DCR), an RNase III endonuclease, with the assistance of a dsRNA-binding protein (Bernstein et al., 2001). DCR cleavage yields an RNA duplex with 5′ phosphates and a two nucleotide overhang on the 3′ end. Guide and passenger strands are determined by the relative thermodynamic stabilities of the 5′ ends, and the guide strand is loaded into the RNA-induced silencing complex (RISC) with an Argonaute (AGO) protein and in some cases auxiliary proteins (Tabara et al., 1999; Hammond et al., 2000; Zamore et al., 2000). RISC complexes containing siRNAs repress their target mRNAs through cleavage by AGO, which leads to the production of secondary siRNAs, a process known as RNA interference (RNAi). Unlike siRNAs, miRNAs do not require an RDR for biogenesis. miRNAs originate from a primary miRNA transcript (pri-miRNA), which forms a secondary stem and loop structure. The mature miRNA is formed through two processing steps by an RNase III endonuclease and dsRNA-binding domain partner protein (Denli and Hannon, 2003). In animals, the pri-miRNA is processed in the nucleus by Drosha, producing a pre-miRNA with a 3′ overhang (Lee et al., 2003). The pre-miRNA is then exported to the cytoplasm, where it undergoes a second processing step by Dicer to form the mature miRNA/miRNA^*^ (e.g., guide/passenger) duplex. The miRNA guide strand, similar to siRNAs, is chosen through thermodynamic selection and loaded into a RISC complex. In plants, DCL1 performs both processing steps of the pri-miRNA in the nucleus (Park et al., 2002; Reinhart et al., 2002; Schauer et al., 2002). The mechanism of miRNA target regulation, by either cleavage or translational repression, is determined by which AGO binds the miRNA and the degree of complementarity to the target mRNA. Although miRNAs have been observed in plants and animals, no conservation of the miRNA genes has been found across kingdoms. Canonical miRNAs have not been isolated from fungi, but a recent study by Braun et al. (2010) described putative miRNAs in *Toxoplasma gondii*, an apicomplexan in the Chromalveolate supergroup. One miRNA family was found to be conserved in all published *Phytophthora* genomes, with evidence for canonical target mRNA cleavage for two predicted targets of the miRNA in *P. sojae* (Fahlgren et al., 2013).

Dicer (DCR) homologs, which include Dicer-like (DCL) proteins, are well conserved across many eukaryotic kingdoms, yet the number of homologs within a taxonomic group is quite variable. Dicers have been studied most extensively in animals, plants, and fungi, and limited numbers of Dicer homologs have been identified bioinformatically in other eukaryote lineages. *Trypanosoma brucei*, from the Excavata supergroup, contains two Dicer-like proteins, although the structure is unusual (Shi et al., 2006). From the Chromalveolate supergroup, diverse numbers of small RNA-related genes have been identified from a limited number of organisms. The apicomplexan *T. gondii* has a complex small RNA landscape, yet contains one homolog each of DCR, RDR, and AGO (Braun et al., 2010). The ciliate *Tetrahymena thermophila* harbors three DCR homologs (Lee and Collins, 2007), while *Paramecium tetraurelia* has seven annotated DCR/DCL homologs, with a proportion of each potentially being nonfunctional (Lepère et al., 2009). Higher metazoans have one Dicer homolog, insects and Cnidarians have two Dicers, and basal animals (Placozoa and Porifera) have up to five Dicers. Phylogenetic analysis of this larger set of metazoans by de Jong et al. (2009) indicates an ancient duplication event of a “Proto-Dicer” gene, with only Placozoans retaining both versions. The multiple Dicers in the basal metazoans are thought to be the result of lineage-specific duplications. Plants have also gained multiple DCL homologs through duplication, and studies of these paralogs reveal differentiation of function, including miRNA and siRNA specificity and some with roles in viral defense (Park et al., 2002; Xie et al., 2004, 2005; Dunoyer et al., 2005; Gasciolli et al., 2005; Kasschau et al., 2007). This suggests that plants use their specialized RNA machinery for immune defense, and may also explain the higher number of Dicer homologs in basal metazoans (de Jong et al., 2009). All Dicers have two RNase III domains (a and b) in the C-terminus. Other domains commonly found include the DEAD-box helicase, helicase C, dsRBD, PAZ, and dsrm. The PAZ, RNase III, and dsrm domains are responsible for dsRNA-binding and cleavage (Liu et al., 2009). PAZ binds the 3′ two-base overhang of dsRNA, and is connected to RNase IIIa by a long α-helix whose distance functions as a molecular ruler to determine the length of the cleaved RNA (MacRae et al., 2006). RNase III domains can also directly bind the PIWI domain of AGO (Tahbaz et al., 2004). The dsRBD is involved in siRNA/miRNA strand selection (Dlakić, 2006).

RDR has also been conserved across eukaryotic kingdoms, although very few animal RDR homologs have been identified, and variation in the number of homologs among different groups is also observed. A study by Cerutti and Casas-Mollano (2006) reconstructed the putative small RNA gene complex of the last common eukaryotic ancestor. Their phylogenetic analyses across five of the eukaryotic supergroups suggested a monophyletic origin for DCR and RDR, while more-specific RNAi-mediated pathways were independently acquired in some lineages and some RNAi pathways or factors were lost in other lineages. For example, an RDR homolog is generally not present in species that can produce dsRNA by other methods. The evolutionary relationships of the RDR tree were not clear, however, potentially due to the use of neighbor-joining trees for analysis (Cerutti and Casas-Mollano, 2006). A more comprehensive analysis by Zong et al. (2009), including plants, fungi, invertebrate animals, and other eukaryotes proposed an alternate model where the eukaryotic ancestor had three copies of RDR: α, β, and γ. Phylogenetic analysis and patterns of conserved protein sequence motifs provided evidence for the three ancient homologs, although the only defined domain common to all RDR homologs was the RNA-dependent RNA polymerase (RDRP) domain. The variability in homolog numbers among different organisms was suggested to result from further duplication events after divergence of the major groups, with the α- and γ-type RDR homologs showing greater expansion. The duplication of RDR homologs, similar to Dicer, has allowed differentiation of function, as has been demonstrated in plants (Dalmay et al., 2000; Yu et al., 2003; Xie et al., 2004; Herr et al., 2005; Kasschau et al., 2007). Although the analysis by Zong et al. (2009) did not result in strong support values for the relationships of other eukaryotic clades to plant, animal or fungal clades, all of the homologs from other eukaryotic groups were clustered within each of the three putative ancient clades.

Previous *Phytophthora* studies have provided evidence for functional RNA interference pathways, both post-transcriptional gene silencing and transcriptional gene silencing. Initial reports of transgene inactivation (Judelson and Whittaker, 1995) were followed by directed transgene and endogenous gene silencing by sense, anti-sense, and promoter-less constructs (van West et al., 1999; Gaulin et al., 2002; Latijnhouwers and Govers, 2003; Blanco and Judelson, 2005), or alternately a hairpin construct to induce silencing of an endogenous gene (Judelson and Tani, 2007). The presence of 21-nucleotide RNAs from both the sense and anti-sense strands of the transgene were observed in partially silenced lines (Ah-Fong et al., 2008). The method of transgene silencing was proposed to initially be through post-transcriptional gene silencing using small RNA, followed by transcriptional gene silencing through chromatin modification (Ah-Fong et al., 2008). The study also bioinformatically identified small RNA-processing enzymes from *P. infestans*, including one Dicer-like (DCL1), one RDR, and five AGO homologs. A study of the expression of these and other possible small RNA-related proteins provided evidence for DCL1 and AGO1,2 playing a role in transgene silencing (Vetukuri et al., 2011). An alternate domain structure of *P. infestans* RDR containing a DCR-like DEAD-box helicase was observed, similar to the structure seen in *Dictyostelium* (Vetukuri et al., 2011). High-throughput sequencing of *P. infestans* small RNAs revealed peaks at sizes of 21- and 25/26-nt (Vetukuri et al., 2012). Additionally, there was evidence for DCL1 having a role in production of 21-nt size small RNAs. This differs from the diatoms *Phaeodactylum tricornutum* and *Thalassiosira pseudonana*, which also have one DCR-like protein, but small RNA sequencing data revealed only one peak of small RNAs at 22-nt (Huang et al., 2011; Norden-Krichmar et al., 2011). In *P. infestans*, similar to *P. sojae* and *P. ramorum*, there are in fact two conserved DCL genes, one RDR, and five AGO genes, with *P. sojae* and *P. ramorum* having additional AGO genes and pseudogenes (Fahlgren et al., 2013). The small RNA peaks at 21- and 25/26-nt were also confirmed for those three species (Fahlgren et al., 2013). One conserved miRNA family was discovered in the three species, and modified 5′ RACE confirmed canonical cleavage of two predicted targets (Fahlgren et al., 2013).

The goal this study was to conduct a comprehensive analysis of the evolution of the DCR and RDR genes in oomycetes. We cloned and annotated the small RNA biogenesis genes *DCL1, DCL2*, and *RDR* in *P. sojae* and analyzed their expression in various life stages. We experimentally validated nuclear targeting of DCL1 and DCL2. We performed phylogenetic analyses of oomycete and other Stramenopile DCLs and RDRs compared to those from a broad range of eukaryotic kingdoms using the key conserved domains of each protein. Domain transfer of the DEAD-box helicase domain from DCR to RDR in oomycetes is also discussed, as well as a proposed function for the two DCR homologs.

## Materials and methods

### Selection of eukaryotic genes for phylogenetic analyses

Dicer and RDR homolog sequences were initially identified in the NCBI database (www.ncbi.nlm.nih.gov) using TBLASTN searches. Various queries were used, including full-length sequences or RNase III/RDRP domain sequences from species such as *Homo sapiens, Trichoplax adhaerens, Arabidopsis thaliana*, and *Phytophthora sojae*. Species with partial or fully-sequenced genomes were selected from all eukaryotic supergroups with the exception of Rhizaria. DCR and RDR homologs identified in previous phylogenetic analyses (de Jong et al., 2009; Zong et al., 2009) were included along with homologs from kingdoms that were less represented previously. Additional sequences were identified through TBLASTN searches of species-specific genome databases, and the DOE JGI database (genome.jgi-psf.org) was searched by querying specific organisms for the presence of annotated RNase III, DEAD-box helicase, or RDRP domains. Research in different organisms resulted in different naming conventions (DCR and DCL); when already annotated we retained the provided name, otherwise we used the DCR designation. Oomycete homolog sequences were identified at the VBI Microbial Database (currently housed at: eumicrobedb.org) and the *P. infestans* database at the Broad Institute (www.broadinstitute.org/annotation/genome/phytophthora_infestans/MultiHome.html). *Peronospora tabacina* homolog sequences were originally provided by David Zaitlin, and the genome has recently been published (Derevnina et al., 2015). Genomic sequences encompassing the identified homologs were isolated and coding sequences were manually identified or confirmed using Genscan (Burge and Karlin, 1997) or FGENESH (linux1.softberry.com/berry.phtml). Protein domains were identified using a search of the Pfam database (URL; Finn et al., 2010). To be retained for subsequent analyses, a Dicer homolog minimally had to contain two RNase III domains, and an RDR homolog minimally had to contain an RDRP domain. Drosha homologs and RNase III domain-containing genes from bacteria and yeast were also identified to provide outgroups. *P. sojae* sequences have been deposited at GenBank under the following accession numbers: DCL1 (KT387801, KT387802), DCL2 (KT387803), and RDR (KT387804).

### Cloning and sequencing of *P. sojae* DCL and RDR homologs

Total RNA was isolated from mycelium of *P. sojae* strain P6497 using TRIzol (Invitrogen, Carlsbad, CA), and mRNA was isolated from 250 μg total RNA with an Oligotex column (Qiagen, Germantown, MD) cDNA was synthesized with the GeneRacer RACE Ready kit (Invitrogen) following the manufacturer's guidelines. Genscan-predicted coding sequences from genome database queries were analyzed with the Primer3 (Rozen and Skaletsky, 2000) plugin in Geneious version 8.0.2 (Kearse et al., 2012) to design primers. Primers were initially tested on genomic DNA isolated from mycelium with a FastPrep kit (MP Biomedicals, Solon, OH) to confirm the predicted genome sequence and test the efficacy of the primers. Primer sequences are listed in Supplementary Table 1. All PCR products were amplified with GenScript Taq polymerase following manufacturer's guidelines: all primers were designed to use annealing temperatures between 56 and 60°C, with an extension time of 1 min per 1 kb. Amplification of the 5′ and 3′ UTRs utilized touchdown PCR, as recommended by GeneRacer guidelines, typically with 1-min extension. Internal sequence amplification typically involved 60°C annealing temperature and 3-min extensions. All PCR products were cloned with the TOPO-TA cloning kit (Invitrogen). A minimum of four clones were sequenced for each product with BigDye Terminator v. 3.1 Cycle Sequencing Kit on an ABI Prism 3730 Genetic Analyzer (Applied Biosystems, Carlsbad, CA) at the Oregon State University Center for Genome Research and Biocomputing. The sequences were assembled and analyzed manually using Geneious software. The full-length *DCL1* and *DCL2* sequences were amplified from *P. sojae* cDNA using primers DCL1-F/DCL1-R (GC buffer and touchdown program from 68 to 58°C) and DCL2-F/DCL2-R (standard manufacturer protocol), and Phusion® High-Fidelity DNA Polymerase (NEB) respectively. The PCR amplicons were in-frame fused with *GFP* by inserting into the blunt site *Stu* I in the plasmid pYF2-GFP (Fang and Tyler, 2016).

### qRT-PCR analysis of gene expression

Total RNA was isolated using TRIzol from *P. sojae* strain P6497 mycelium, zoospores, and germinated cysts prepared in six independent biological replicates. Zoospores were produced by repeated washing of 11 day-old V8-200 plates of mycelium followed by incubation overnight at 14°C (Tyler et al., 1996). Germinated cysts were produced from zoospores by exposure to cleared V8 medium for 1 h. Soybean infection experiments followed the protocol of Qutob et al. (2000) in which mycelium-inoculated hypocotyls were incubated at 28°C for 14 h in the light and 25°C for 10 h in the dark. 50 μg of TRIzol-isolated RNA from each replication were purified from DNA using the AllPrep DNA/RNA Mini kit (Qiagen) following the manufacturer's guidelines, with the addition of one DNase I treatment. cDNA was produced using the Superscript III First-Strand Synthesis System for RT-PCR kit (Invitrogen) and purified by phenol:chloroform extraction. cDNA was quantified with a Nanodrop ND-1000 (Thermo Scientific) and equal quantities of cDNA template were added for each reaction. Primers were designed as described above. Standard quantitative real-time PCR (qRT-PCR) reactions were performed with Fast SYBR Green Master Mix (Applied Biosystems) on an ABI StepOnePlus Real-Time PCR System (Applied Biosystems). Data were normalized to reference genes β-Tubulin (Ps109498) and WS41 (Ps137777; protein of the BAR-domain family) and analyzed with Bestkeeper (Pfaffl et al., 2004), REST (Pfaffl et al., 2002), and SAS software (Cary, NC).

### Subcellular localization of *P. sojae* DCL homologs

Laser scanning confocal microscopy (Zeiss LSM 780) was used to examine the expression and subcellular localization of DCL1-GFP and DCL2-GFP fusions. Living hyphae were picked from liquid V8 cultures after 2-3 days growth. Samples were stained with DAPI (4′, 6-diamidino-2-phenylindole) for 20 min in the dark before microscopic examination (Hardham, 2001). Images were captured using a 63X oil objective with excitation/emission settings (in nm) 405/410-490 for DAPI, and 488/510-535 for GFP. Images were edited by Adobe Photoshop CS4. The tonal range was increased by adjusting highlights and shadows without altering the color balance; all images were adjusted identically.

### Phylogenetic analyses

All protein sequences used are provided in Supplementary Data Sheet 7. Alignments of protein sequences were created with MAFFT (mafft.cbrc.jp/alignment/server). For broad species analyses, protein sequences of specific conserved domains were aligned as follows: individual RNase IIIa and RNase IIIb domains, RNase IIIa and RNase IIIb domains concatenated with linking sequences deleted, DEAD-box helicase domains, and RDRP domains. To reduce errors introduced by the presence of partial sequences and to moderate the computational load, amino acid positions which were absent in a majority of the analyzed species were removed from the alignment (Baurain et al., 2010). Drosha homologs were included in both RNase III analyses, as they share the same double domain structure as Dicer homologs. Bacterial ribonuclease (Rnc), *Saccharomyces castellii* DCR1 (Drinnenberg et al., 2009), and *Neurospora crassa* MRPL3 (Lee et al., 2010) each contain only one RNase III domain, and so were limited to the single-domain comparison analysis. Nomenclature of the identified genes followed Zong et al. (2009). Species are identified in the phylogenetic trees with an abbreviated name in the format “Gens” (the first three letters of the genus name followed by the first letter of the species name). Gene number designations do not necessarily follow orthology. Previous designations, either from the genome database or a previous publication (de Jong et al., 2009; Zong et al., 2009), were maintained; otherwise the number assignment was arbitrary when there was no previous reference.

Condensed domain alignments were used for Bayesian phylogenetic analyses using MrBayes-3.1.2 (Huelsenbeck and Ronquist, 2001; Ronquist and Huelsenbeck, 2003). All analyses shared common settings: mixed amino acid model of evolution with invariant gamma rates, unconstrained branch lengths, sample frequency of 50, 2 runs with 4 chains each, temperature of 0.2, and diagnostic frequency of 1000. The concatenated RNase IIIa/b analysis ran for 32 million generations with a final burn-in of 25%, the RNase III individual domain analysis ran for 32 million generations with a final burn-in of 75%, the DEAD-box helicase analysis ran for 32 million generations with a final burn-in of 75%, and the RDRP analysis ran for 32 million generations with a final burn-in of 75%. Conservation plots were produced from the condensed DEAD-box helicase domain MAFFT alignment using Geneious software (Biomatters; www.geneious.com).

## Results

### Cloning and annotation of *P. sojae* DCR and RDR homologs

**Figure 1:**
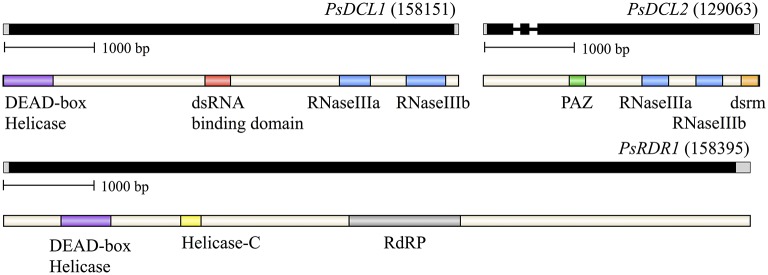
**Organization of ***P. sojae*** small RNA biogenesis genes and proteins, dicer-like (DCL) and RNA-dependent RNA polymerase (RDR)**. Genes are labeled followed by their Gene ID from the EuMicrobeDB Database (eumicrobedb.org). In the genomic DNA diagrams, exons and introns are represented as black bars and lines, respectively. 5′ and 3′ UTRs are represented as gray bars. Conserved domains are indicated by colored bars in the mRNA diagrams: dsrm, dsRNA binding motif; RdRP, RNA-dependent RNA polymerase domain; PAZ, PAZ domain named for the proteins Piwi Argonaute and Zwille.

*P. sojae* homologs of DCR and RDR were initially identified bioinformatically from the VBI Microbial Database (currently housed at: eumicrobedb.org). We isolated cDNA from *P. sojae* mycelium and amplified overlapping segments across the length of each gene, including the use of 5′ RACE and 3′ RACE, to confirm the predicted gene structures and annotate the 5′ and 3′ UTR regions (see Supplementary Figure 1 for primer map and Supplementary Table 1 for primer information). Figure 1 shows the annotated gene structures along with conserved protein domains as predicted by Pfam. All three genes produce short UTRs: the 5′ UTRs ranged from 32 to 52 bases in length, and the 3′ UTRs ranged from 35 to 140 bp in length. Of the three annotated genes, *DCL2* contains two introns that are 71 and 83 bases in length. The GC content of the transcribed sequences is 55.2% (*DCL1*), 55.6% (*DCL2*), and 52.2% (*RDR*). Thus the genes follow the general trends of *Phytophthora* genes, where only one-third possess an intron (average length of 79 bases), of which the average is 1.5 introns per gene, and transcribed sequences have a higher GC content of 58% (Kamoun, 2003). The sequences surrounding the transcription start site, translation start site, and exon/intron boundaries also generally follow the trend seen in other *Phytophthora* genes (Supplementary Figure 2).

All but one of the commonly observed Dicer protein domains are present in the *P. sojae* homologs, although only the two RNase III domains are present in both DCL1 and DCL2. DCL1 additionally contains the DEAD-box helicase and dsRBD domains, whereas the PAZ and dsrm domains are found only within DCL2, although the PAZ domain is less well conserved and harder to predict. In comparison, Pfam predicted domains in the DCL homologs from plants harbor almost all common domains in each homolog. This suggests the *P. sojae* homologs may have a differentiation of function in the two DCL loci more diverged than among the plant homologs. For all phylogenetic analyses presented, Pfam predicted domain structures are presented for each homolog included in the analysis to allow for comparison of domain structure along with phylogenetic placement.

### Gene expression of *DCL1, DCL2*, and *RDR*

To ascertain if *P. sojae DCL1, DCL2*, and *RDR* are functional loci and expressed in various life stages, gene expression was assessed with qRT-PCR analysis. All three genes were actively transcribed in all examined tissues at a level 15- to 150-fold lower than the control genes β-Tubulin (Ps109498) and WS41 (Ps137777; protein of the BAR-domain family), and only small changes in relative expression levels were detected (Supplementary Figure 3). The only life stage samples that were significantly different than mycelium were zoospores (2.2 to 3.6-fold elevation in transcript levels) and DCL1 expression in germinated cysts (6.8-fold decrease in transcript levels; 1.9–2.9 decrease in DCL2 and RDR transcript levels was not significantly different than mycelium). It is unknown if these changes in expression would have a significant impact on small RNA levels.

### *P. sojae* DCL1 and DCL2 are nucleus localized

**Figure 2:**
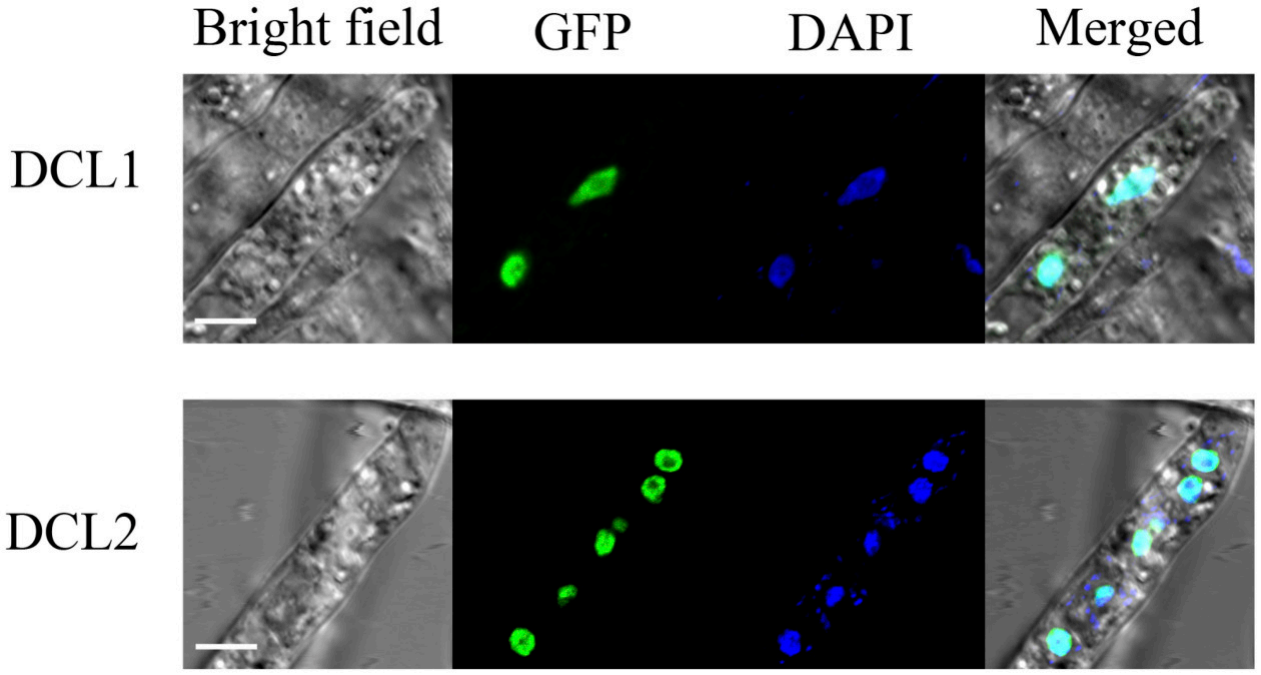
**Nuclear localization of DCL1 and DCL2 in ***P. sojae*** hyphae**. Subcellular localization of both GFP tagged dicers overlaps with the nuclear marker DAPI. Scale bar, 5 μm.

Based on nuclear localization signal (NLS) prediction using pSORT II (Nakai and Horton, 1999), there were a few candidate NLS found in *P. sojae* DCL1, while no NLS were predicted in DCL2. NLS have not been defined functionally in *P. sojae*, and several classical NLS worked poorly in *P. sojae* (Fang and Tyler, 2016). To determine the subcellular localization of *P. sojae* DCL1 and DCL2 directly, the two full-length genes were amplified and fused at the C termini with green fluorescent protein (GFP). The fusion genes were then introduced into *P. sojae* transformants. Examination of three independent *P. sojae* transformants overexpressing DCL1-GFP or DCL2-GFP by confocal microscopy revealed that both Dicers showed strong nuclear localization (Figure 2, Supplementary Figure 4).

### Phylogenetic analyses of the RNase III domains

**Figure 3:**
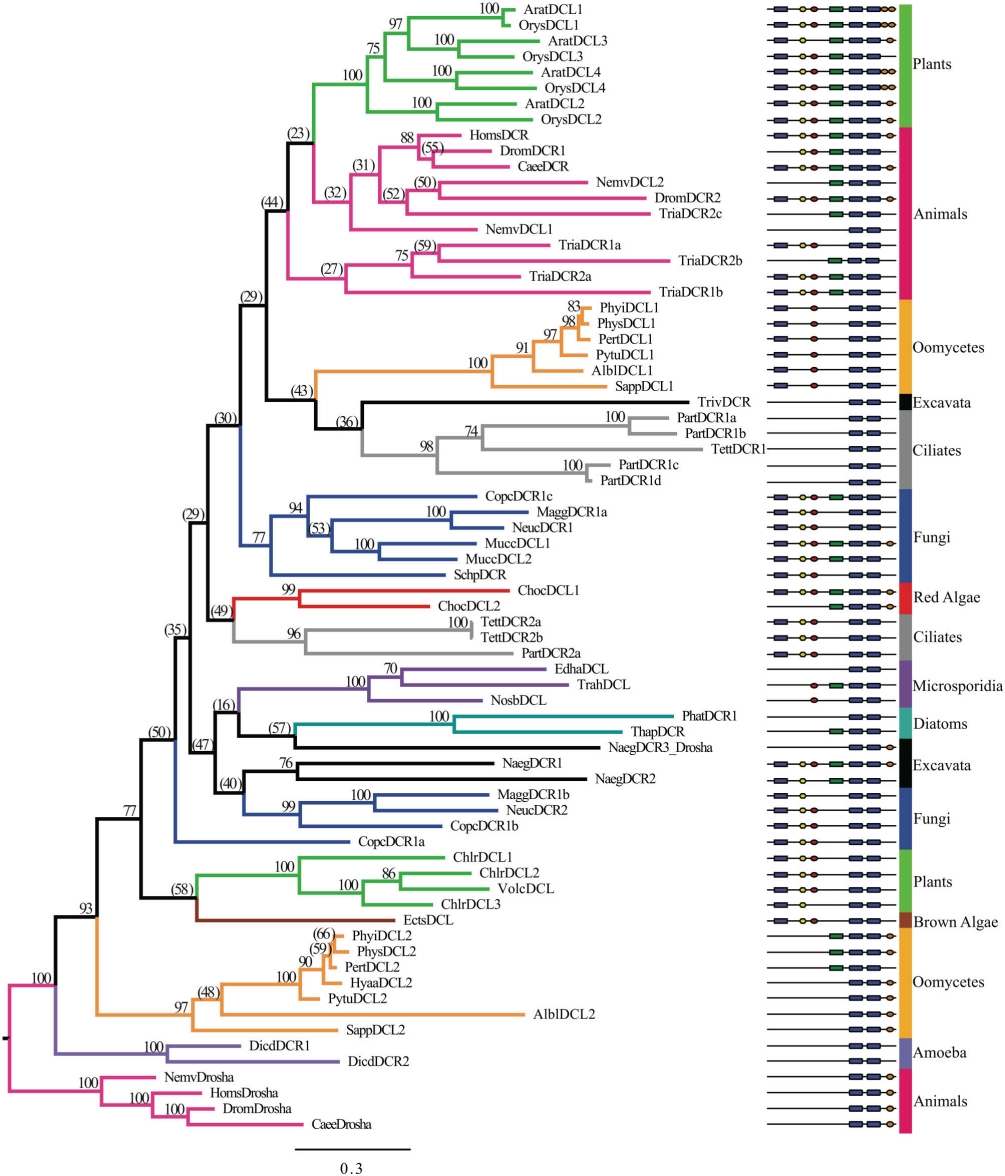
**Phylogenetic tree of Dicer homologs based on the concatenated RNase IIIa and RNase IIIb domains**. Bayesian support values are shown next to nodes. Branches are colored to denote major species groups, as labeled on the right. Gene model diagrams (not drawn to scale) indicate Pfam predicted domains: left to right: purple rectangle, DEAD-box helicase; yellow hexagon, helicase-C domain; red oval, dsRBD; green rectangle, PAZ domain; blue rounded boxes, RNase IIIa and RNase IIIb domains; orange oval, dsrm. Species abbreviations are defined in Supplementary Table 2A.

A broad spectrum of eukaryotic species was selected to analyze the evolutionary origins of oomycete DCR homologs. In all, 72 DCR homologs were identified in 33 species, as listed in Supplementary Table 2A. Research in different organisms resulted in different naming conventions (DCR and DCL); when already annotated we retained the provided name, otherwise we used the DCR designation. The number of homologs present from each analyzed species varied. Plants had three to five DCL homologs, in contrast to most animals, which harbored one to two Dicers and one Drosha. Chromalveolates varied greatly between subgroups, for example oomycetes contained two DCL homologs and ciliates contained varied numbers of homologs. Two separate analyses were performed to elucidate the origins of the oomycete DCR homologs. In the first analysis (Figure 3, Supplementary Data Sheet 3), the amino acid sequences from the RNase IIIa and RNase IIIb domains of the DCR and Drosha homologs were concatenated to study the overall evolution of the key catalytic domains. The RNase III domains were selected rather than the full length protein sequences due to the great diversity of species included in the analysis. As expected, Drosha homologs formed an outgroup to DCR homologs. The two families of oomycete DCR homologs formed two distinct well-separated clades. The DCL1 clade was associated with a poorly separated set of clades that included ciliates, higher plants, animals and fungi. The DCL2 homologs formed a much more basal clade with some affinity to green and brown algal DCL's.

**Figure 4:**
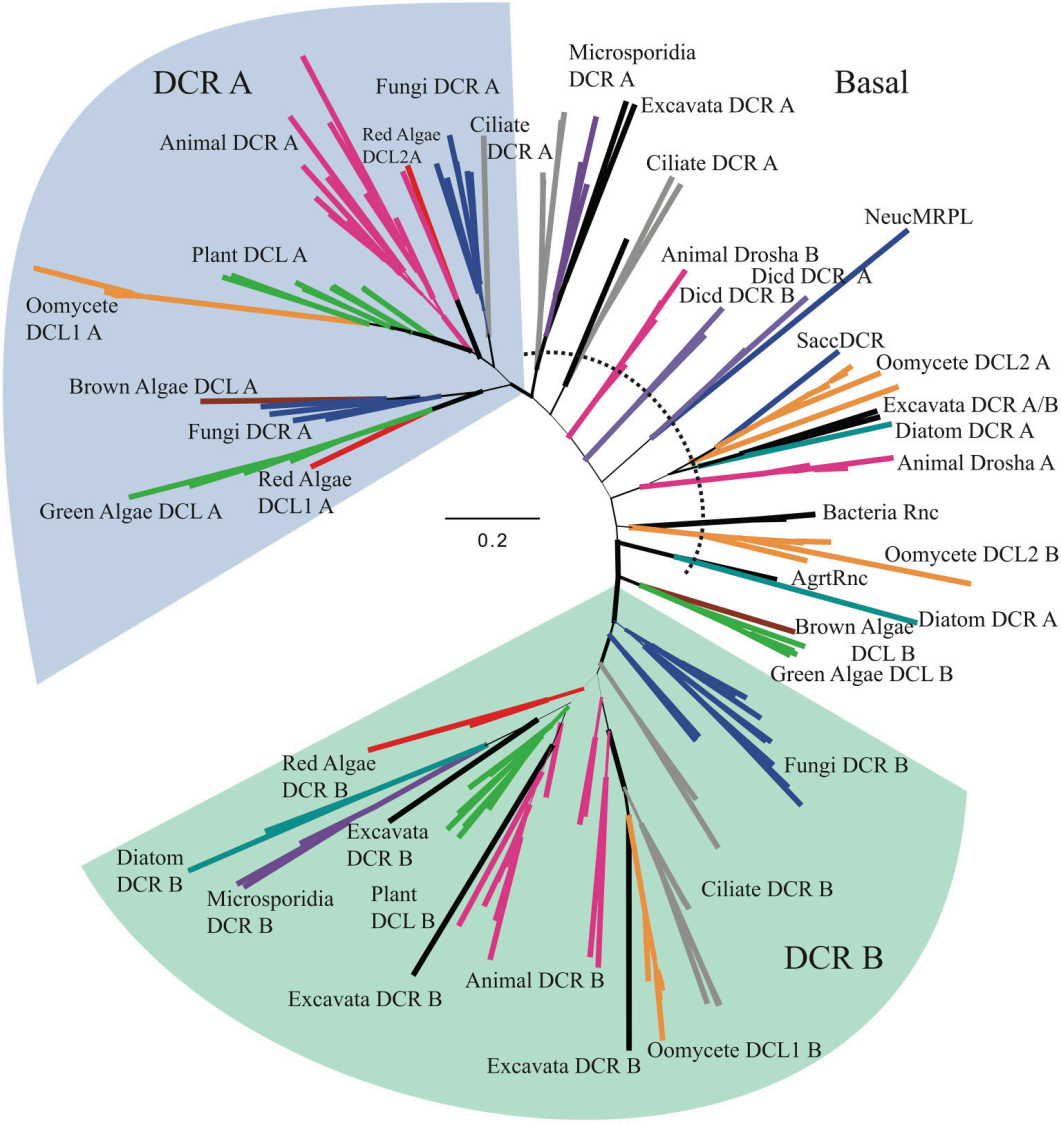
**Radial, phylogenetic tree of Dicer homologs based on individual RNase IIIa and RNase IIIb domains**. Branch thicknesses signify significance of Bayesian support. Branches are colored to denote major species groups, as in Figures 2, 4, and 5. Species abbreviations are defined in Supplementary Table 2A.

To further investigate the basal placement of DCL2, a second analysis (Figure 4, Supplementary Figures 5A,B, Supplementary Data Sheet 4) was performed comparing the RNase IIIa and RNase IIIb (indicated in the Supplementary Figures 5A,B, respectively) domains of all DCR and Drosha homologs individually to enable more detailed analysis of this domains' evolution. Six additional sequences from bacterial and yeast ribonucleases, which only contain a single RNase III domain, were included to provide more diversity in the tree, especially at basal levels. The RNase IIIa domains of oomycete DCL1 homologs clustered with higher plant RNase IIIa within the DCR A clade, while the RNase IIIb domains clustered with ciliate and animal homologs within the DCR B clade.

The analysis of the RNase III domains of the oomycete DCL2 homologs revealed that the two domains formed distinct clusters within a poorly resolved set of basal clades that includes two Drosha clades. The DCL2 RNase IIIa domains showed weak affinity with some diatom, excavate and yeast sequences, while the RNase IIIb domains showed weak affinity with bacterial Rnc sequences. The RNase III domain from the budding yeast DCR (*SaccDCR*), which has recently been described as a noncanonical Dicer that may function as a homodimer in an endogenous siRNA pathway (Drinnenberg et al., 2009), clusters tightly with the oomycete DCL2 RNase IIIa domain. Therefore the four RNase III domains of the oomycete DCRs all show widely different phylogenetic affinities.

While all the oomycetes tested, from *Phytophthora* to *Saprolegnia*, shared a common set of DCR homologs with oomycete-specific origins, the other Stramenopiles tested only had a single DCR homolog that had a different origin. For the brown algae *Ectocarpus siliculosus*, both the analysis of the combined RNase III domains and the separated domains showed the sequences clustering with homologs from green algae. For the diatoms *P. tricornutum* and *T. pseudonana*, the combined analysis placed the DCR homologs within a poorly resolved set of clades including excavates, microsporidia, and fungi (Supplementary Figure 5). The separated analysis showed unexpectedly that the RNase IIIa domains of the two diatom DCRs showed very different affinities. The *T. pseudonana* sequence clustered with the excavates and the oomycetes, while the *P. tricornutum* clustered with a bacterial Rnc (from *Agrobacterium tumefaciens*). On the other hand, the two RNase IIIb domains clustered tightly together, with affinity to microsporidial sequences.

### Phylogenetic analysis of the DEAD-box helicase domain

Analysis of conserved domain structures in oomycete small RNA-processing proteins confirmed the presence of a putative DEAD-box helicase domain in the DCL1 homologs, but not within the DCL2 homologs (e.g., Figure 1). The amino terminus of the RDR homolog also contained a DEAD-box helicase domain, together with a related helicase-C domain. This domain arrangement has been reported previously in *Dictyostelium discoideum* (Martens et al., 2002; Kuhlmann et al., 2005; Hinas et al., 2007), but not in other organisms. In addition to the oomycete and *D. discoideum* RDR homologs, we identified the same DEAD-box helicase—helicase-C—RDRP domain structure in RDR homologs from the lower animals *Hydra magnipapillata* and *Nematostella vectensis*. As reported in *D. discoideum* (Martens et al., 2002) and as determined bioinformatically for *H. magnipapillata* and *N. vectensis*, the altered RDR structure is coincidental with loss of DEAD-box helicase domains in each organisms' DCR homologs.

**Figure 5:**
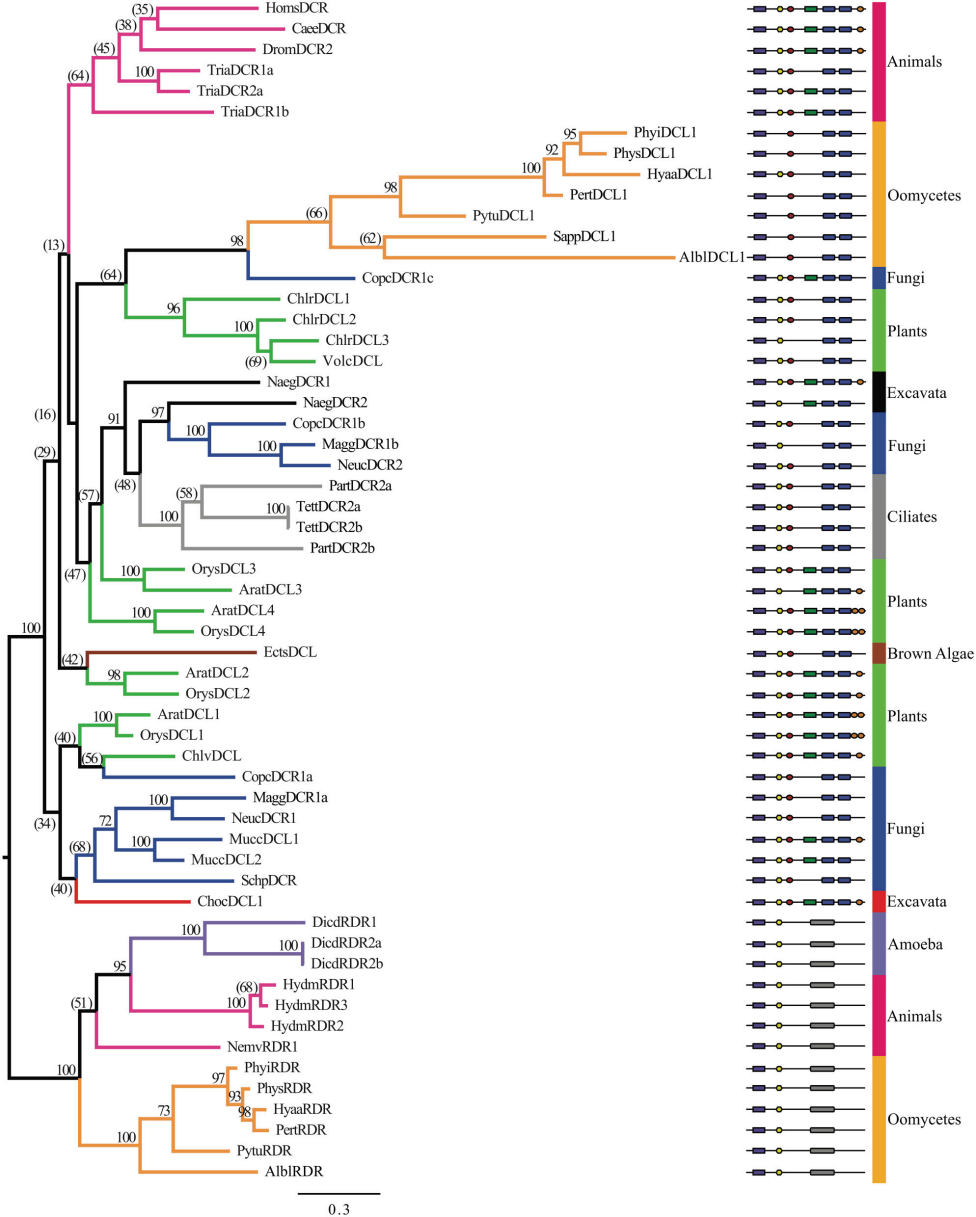
**Phylogenetic tree of Dicer and RDR homologs based on the DEAD-box helicase domain**. Bayesian support values are shown next to nodes. Branches are colored to denote major species groups, as labeled on the right. Gene model diagrams (not drawn to scale) indicate the Pfam predicted domains: left to right: purple rectangle, DEAD-box helicase; yellow hexagon, helicase-C domain; red oval, dsRBD; green rectangle, PAZ domain; blue rounded boxes, RNase IIIa and RNase IIIb domains; orange oval, dsrm; gray rounded box, RdRP domain. Species abbreviations are defined in Supplementary Table 2A.

DEAD-box helicase sequences from both DCR and RDR homologs were analyzed in a single tree to elucidate the potential origins of the oomycete sequences. Figure 5 (Supplementary Data Sheet 5) displays the phylogenetic analysis of the DEAD-box helicase domains. The DEAD-box helicases from RDR homologs showed a strong separation from the DEAD-box helicase domains from the DCR homologs, indicating distinct evolutionary origins. The DCR clade contained a large number of poorly resolved subclades corresponding to different kingdoms. The oomycete DCL1 sub-tree showed exceptionally long branch lengths, suggesting rapid divergence of the sequences within the oomycetes. Interestingly, the RDR DEAD-box helicase domains from oomycetes, *Dictyostelium, Hydra*, and *Nematostella* clustered together and were strongly separated from all the DCL1 dead box domains.

A reciprocal best-blast analysis was performed to determine if there were potential orthology relationships between the DEAD-box helicase-containing RDR homologs. The DEAD-box helicase domains from *D. discoideum* and *H. magnipapillata* RDR homologs were reciprocal best-blast hits, suggesting a common evolutionary origin at the base of the animal and amoebozoa kingdoms. However, the DEAD-box helicase domains from the oomycete RDR homologs showed no consistent affinity with other evolutionary groups, including animal and amoebozoa RDR homologs or with the oomycete DCL1 homologs.

### Phylogenetic analysis of the RDRP domain from RDR homologs

**Figure 6:**
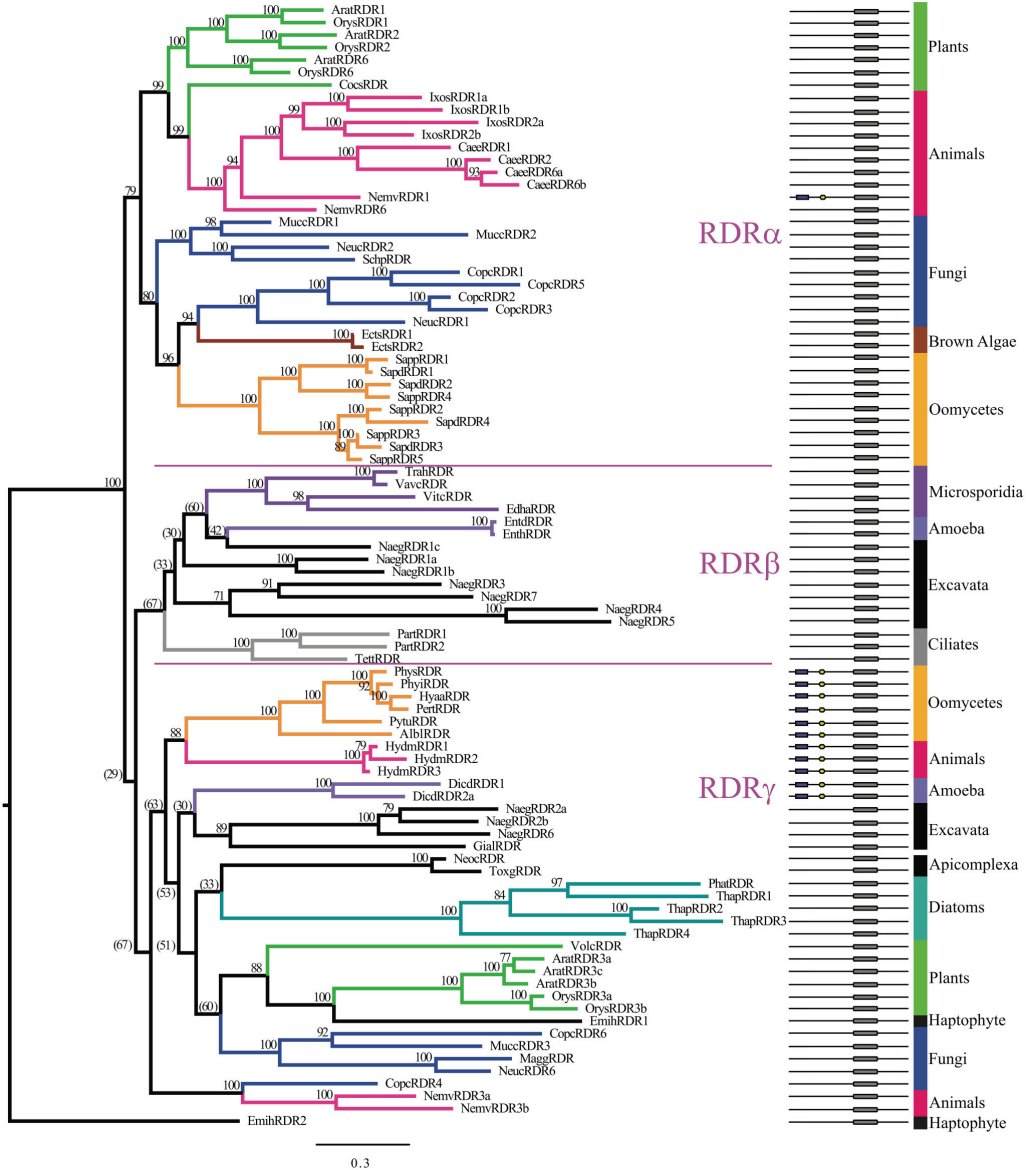
**Phylogenetic tree of RDR homologs based on the RdRP domain**. Bayesian support values are shown next to nodes. Branches are colored to denote major species groups, as labeled on the right. Gene model diagrams (not drawn to scale) indicate the Pfam predicted domains: left to right: purple rectangle, DEAD-box helicase; yellow hexagon, helicase-C domain; gray rounded box, RdRP domain. The three ancestral RDR clades (α, β, γ) are labeled in reference to the study by Zong et al. (2009). Species abbreviations are defined in Supplementary Table 2B.

To refine the origin of oomycete RDR homologs as examined by Zong et al. (2009), we included additional oomycete, Stramenopile, protist, and basal metazoan sequences to broaden the phylogenetic analysis. In all, 90 RDR homologs were identified in 38 species, as listed in Supplementary Table 2B. Similar to DCR homologs, the number of homologs present for each species analyzed varied. Plants had five to six RDR homologs, whereas most animals did not have RDR homologs at all. A small number of lower animals, including *Ixodes scapularis, Branchiostoma floridae*, nematodes, and cnidarians contained multiple RDR homologs, ranging from 3 to 7. Stramenopiles varied greatly between subgroups. For example, oomycetes from *Phytophthora* to *Albugo* contained only one RDR homolog, but *Saprolegnia* contained up to five homologs, and diatoms contained a varied numbers of homologs. Phylogenetic analysis of the RDRP domain (Figure 6, Supplementary Data Sheet 6) confirmed the overall tree published by Zong et al. (2009) with some changes. In the previous analysis, the oomycete homolog (*P. ramorum*) was basal to the RDRα animal clade. The current study refined the placement of *Phytophthora, Hyaloperonospora, Peronospora, Pythium*, and *Albugo* homologs as basal to the RDRγ clade along with *Hydra* homologs, while the *Saprolegnia* and *Ectocarpus* homologs clustered with fungi in the RDRα clade (Figure 6, bottom). The occupants of the RDRβ clade were also clarified, with ciliate homologs basal to the clade. Some fungal and animal sequences were located in the RDRγ cluster, but others did not associate clearly with either clade β or clade γ. The larger number of generations in the current analysis (32 million versus 1 million) allowed better refinement of the overall tree, with each major RDR group showing fairly good support for a tree with this diverse range of species. The separation of primarily two main clades with a high Bayesian posterior probability of 100 only partially supports the previous study's hypothesis that there were three RDR homologs in the eukaryotic ancestor (Zong et al., 2009). In our analysis, the Bayesian posterior probability of separation of the β and γ clades was only 29%.

### Conservation analysis of key residues

To analyze the potential for the oomycete DCR and RDR homologs to possess their canonical functions, conserved residues shown to be important for catalytic activity were compared within the domain alignments (Supplementary Figures 6–8). For the RNase III domain (Supplementary Figure 6), catalytic residues ExxGD and DxxE were found at the beginning and end of the domain, respectively (Lepère et al., 2009). For the RDRP domain (Supplementary Figure 7) the key residues are DLDGD, with the last aspartic acid required for catalytic activity (Lee and Collins, 2007; Marker et al., 2010). Manual analysis of the key catalytic residues in the RNase III and RDRP domains of the oomycete DCR and RDR homologs showed perfect conservation of the consensus sequence for the majority of the homologs. Variations in consensus sequence in the RNase III domain included: all four Drosha RNase IIIa homologs had NxxE in the second motif, *T. adhaerens* DCL1a RNase IIIa, *Naegleria gruberi* DCR3 RNase IIIa, *P. tricornutum* DCR1 RNase IIIa, and *Albugo laibachii* DCL2 RNase IIIb had variation at one or both residues of the second motif, *Naegleria gruberi* DCR3 RNase IIIb had KxxxS in the first motif, and *T. adhaerens* DCL1b RNase IIIa, *T. adhaerens* DCL2b RNase IIIb, *P. tricornutum* DCR1 RNase IIIb, *T. pseudonana* DCR RNase IIIb, *Albugo laibachii* DCL2 RNase IIIa, and *Neurospora crassa* MRPL3 had variations in both motifs. Variations in the consensus sequence in the RDRP domain were mostly limited to the second residue (making it DxDGD). Only one homolog with a complete sequence covering that region had more than the change at the second residue—*Mucor circinelloides* RDR2 (DPDED). In the case of *Albugo laibachii* RDR (–DSE), the sequence is not clear at that site due to unresolved intron placement. More significant deviations from consensus sequences were observed, however, in the DEAD-box helicase domain (Supplementary Figure 8), especially in the oomycete DCL1 homologs. Whereas oomycete RDR homologs show conservation of the key site residues GxGKT (ATP-binding residues) and DE[C/A]H (Mg^2+^-binding residues) observed in canonical DEAD-box helicase domains from DCR homologs, the oomycete DCL1 homologs had numerous substitutions in both sequences. Another commonly observed variation was DEVH at the second motif, seen in *Dictyostelium* RDR homologs and *Chondrus crispus* DCL1. Supplementary Figure 9 compares the sequences of the key motifs of the DEAD-box helicase domains among different Stramenopile homologs.

## Discussion

In contrast to other housekeeping genes, the genes encoding the small RNA machinery of eukaryotes display a remarkable degree of diversity, not only in primary sequences, but also in terms of domain structure, and gene family size. Currently the evolutionary forces that drive this diversity, and the mechanisms by which the diversity has been generated remain unknown. While the small RNA machinery of plants, animals and fungi has been quite well characterized, the small RNA machinery of the Stramenopile kingdom, which includes both photosynthetic algae and destructive oomycete pathogens, has received little attention. Here, beginning with the validation of gene models using amplified cDNAs from *P. sojae*, we have demonstrated that the DCL and RDR proteins of oomycetes display unusually distinctive evolutionary origins.

In contrast to other species, such as plants, whose multiple DCR homologs are the result of gene duplication after splitting of the major group lineages, the two DCR homologs in oomycetes have widely divergent origins. Oomycete DCL1 homologs are part of a poorly resolved clade including ciliates, plants, animals, and fungi. This group is well separated from oomycete DCL2 homologs, which resolved more basally in the tree along with *Dictyostelium* DCR and animal Drosha homologs, although these three groups are well separated from each other. This pattern appears in both RNase III domain analyses. The oomycete DCL1 RNase IIIa domain is more plant-like, while the DCL1 RNase IIIb is more similar to ciliates and basal animals. The DCL2 RNase III domains are both more basal. The DCL2 Rnase IIIa domain is similar to yeast, excavate, and diatom RNase domains and very weakly to Drosha RNase IIIa domains while the RNAse IIIb domain is more similar to bacterial Rnc domains. The structural differences between oomycete DCL2 and animal Drosha homologs provide further support for a different function for these proteins. Although both DCR and Drosha homologs contain two RNaseIII domains, a hallmark of Drosha has been the lack of a PAZ domain, which is critical for proper DCR processing of small RNA duplexes. In contrast, the oomycete DCL2 protein has a conserved PAZ domain, suggesting its function to be more DCR-like than Drosha-like. We hypothesize that the functions of DCL1 and DCL2 have independent evolutionary origins and thus must have different functions given the differences in domain content (Figure 1). This is in line with the previously observed support for two distinct small RNA classes of predominantly 21- or 25-nt in length (Fahlgren et al., 2013) that might be processed each by one of the two DCL proteins.

The localization of both oomycete DCL1 and DCL2 homologs to the nucleus does not help to resolve their function. For comparison, *A. thaliana* DCL1 and DCL2, which are responsible for miRNA and viral siRNA pathways, respectively, have predicted NLS sequences, and DCL3 and DCL4, which are responsible for endogenous siRNA and tasiRNA pathways, respectively, do not have predicted NLS sequences. The Drosha homolog from *H. sapiens*, which processes pri-miRNA in the nucleus, has a predicted NLS sequence, but the DCR homolog does not. In other organisms, miRNA processing usually occurs in the nucleus and siRNA processing usually occurs in the cytoplasm, yet in *Phytophthora* it appears that the 21- and 25-nt processing pathways both must occur in the nucleus. The small specks observed in our images may be evidence of cajal bodies in *Phytophthora* nuclei, which could be the centers of miRNA- and siRNA-processing. We cannot rule out the possibility of partial or temporary localization of either DCL in the cytoplasm, as the signal would be diluted and not readily detectable. However our images support predominant nuclear localization of both DCLs.

The origin of the DEAD-box helicase domain in oomycete RDR homologs is not clear. RDR homologs with DEAD-box helicase domains were relatively uncommon among the eukaryote genomes examined. Among the oomycetes, only species in the class *Peronosporomycetidae* (*Phytophthora, Pythium*, downy mildew, *Albugo*) had this arrangement; those from *S. parasitica* (*Saprolegniomycetidae*) did not. Among other eukaryotes, only RDRs from *Dictyostelium* (*Amebozoa*), and from *Hydra* and *Nematostella* (*Animalia*) had this arrangement. The DEAD box domains from all of the RDR homologs, including the peronosporomycetes, formed a single clade separated with 100% support from the DEAD box domains of the Dicer homologs. This observation suggests that the RDR-associated DEAD box domains have an ancient common origin that is distinct from the Dicer DEAD box domains. This inference is supported in part by the observation that the peronosporomycete and *Hydra* RDRs also form a cluster within the RDRγ subgroup based on the RDRP domains of the proteins. The *Dictyostelium* and *Nematostella* RDRs also fall within the RDRγ subgroup, but do not cluster unambiguously with the peronosporomycete and *Hydra* RDRs. If the DEAD box domain-containing RDRs all have a common origin, then either the DEAD box domains must have been lost from some RDRs (e.g., in the RDRγ subgroup) or the DEAD box-containing RDRs may have been entirely lost from some eukaryotic lineages (e.g., those containing no γ subgroup RDRs).

The presence of DEAD box domains in the peronosporomycete, *Dictyostelium* and *Nematostella* RDRs coincides with the loss of DEAD box domains from a subset of the Dicer homologs encoded in each of those genomes (DCL2's in the case of the peronosporomycetes). However, the significance of this coincidence is not clear as in *Saprolegnia* DEAD box domains are absent from its DCL2 as well as all its RDRs. Furthermore, many other eukaryotes that contain Dicer homologs lacking a DEAD box domain also lack RDRs with DEAD box domains.

Of the organisms that contain a DEAD-box helicase domain in the RDR homolog, peronosporomycetes are unique in that they also have a DEAD-box helicase domain in one of the Dicer homologs (i.e., in their DCL1's); *Dictyostelium, Hydra* (not shown), and *Nematostella* only have Dicer homologs that lack DEAD box domains. Furthermore the branch lengths of the DEAD box domains of the peronosporomycete DCL1's are unusually long, whereas the branch lengths of the DCL1 RNase III domains are not exceptionally long. Whether this indicates the DCL1 DEAD box domains are currently degenerating or evolving a novel function is unclear. The oomycete DCL1 homologs are also unique in that they are the only DEAD-box domain-containing proteins that lack a predicted Helicase-C domain. Although the oomycete DCL1 sequences are highly divergent from other DCR homologs, it is unclear what the effect of these changes would be on the catalytic activity of the domain. In the DE[C/A]H box sequence, for example, switching the aspartic acid and glutamic acid residues may not have a huge impact on function, and a tryptophan in the third, most variable, position has unknown significance. As well, even though the other DEAD-box helicase domains in the analysis have a histidine in the fourth position, other members of the helicase family contain aspartic acid in the fourth position. Therefore the oomycete DCL1 homologs could potentially retain their function. Without biochemical or mutagenesis studies it is unclear whether the divergence observed for the DEAD-box helicase is related to function of the domain.

In *D. discoideum*, Martens et al. (2002) suggested that the alternative DEAD box domain arrangement indicated that cleavage of dsRNA and amplification of the signal and guide RNAs are spatially linked, and therefore that the placement of the domain was not critical to function. A study of the DEAD-box helicase from *H. sapiens* DCR suggested the domain aided in the processing of thermodynamically unstable dsRNA structures, such as miRNA precursors, and restricted the processivity of DCR on perfectly-matched substrates (Soifer et al., 2008). It is not clear though how these activities would facilitate RDR function. In *H. sapiens* there is no RDR homolog at all, so the DEAD-box helicase may serve a different role in species that possess RDR homologs. In *Dictyostelium*, the DEAD-box helicase-containing RDR homolog is implicated in the production of ~23-nt siRNAs (Martens et al., 2002). In our phylogenetic analyses, both DCL2 and RDR from *P. sojae* and *Dictyostelium* clustered together, suggesting their similarity of domain structure might also indicate a similarity of function. We speculate that oomycete DCL2 could be involved in a nuclear-localized siRNA/25-nt small RNA pathway.

In the analysis of the DCR A subgroup of RNase III sequences, the oomycete sequences clustered significantly more tightly with plant and animal sequences, and more distantly from fungal sequences, despite the closer evolutionary relationship between animals and fungi. We note that plants, animals and oomycetes (Fahlgren et al., 2013) have miRNA pathways, whereas canonical miRNAs have yet to be discovered in fungi. Thus we speculate that in oomycetes DCL1 could be involved in the miRNA/21-nt small RNA pathway. This is supported by evidence from Vetukuri et al. (2012), who showed that partial silencing of DCL1 in *P. infestans* led to decreased production of assayed 21-nt small RNAs.

Work is currently underway to analyze the small RNA landscape in *P. sojae* through high-throughput sequencing of small RNA from various life stages and infection time points. Ultimately, a better understanding of oomycete small RNA pathways may lead to improved techniques for silencing genes of interest or discovery of small RNA pathways related to host-pathogen interactions.

## Author contributions

SB, YF, CP, BT, and NG all contributed to designing and analyzing experiments, specifically SB was the primary contributor with help from CP and NG on gene expression design and NG and BT on phylogenetic and conservation analysis; YF designed and performed the localization experiments with guidance from BT. All authors also contributed to the drafting, revising, and final approval of the manuscript and are accountable for its accuracy.

## Funding

This work was supported by the USDA National Research Initiative (grant number 2008-35600-18780); and the USDA Agricultural Research Service (CRIS number 5358-22000-03900D).

### Conflict of interest statement

The authors declare that the research was conducted in the absence of any commercial or financial relationships that could be construed as a potential conflict of interest.

## References

[B1] Ah-Fong A. M. V. Bormann-Chung C. A. Judelson H. S. (2008). Optimization of transgene-mediated silencing in *Phytophthora infestans* and its association with small-interfering RNAs. Fungal Genet. Biol. 45, 1197–1205. 10.1016/j.fgb.2008.05.009 18599326

[B2] Alemdehy M. F. Haanstra J. R. de Looper H. W. J. van Strien P. M. H. Verhagen-Oldenampsen J. Caljouw Y.. (2015). ICL-induced miR139-3p and miR199a-3p have opposite roles in hematopoietic cell expansion and leukemic transformation. Blood 125, 3937–3948. 10.1182/blood-2014-11-612507 25778535

[B3] Aravin A. A. Naumova N. M. Tulin A. V. Vagin V. V. Rozovsky Y. M. Gvozdev V. A. (2001). Double-stranded RNA-mediated silencing of genomic tandem repeats and transposable elements in the *D. melanogaster* germline. Curr. Biol. 11, 1017–1027. 10.1016/S0960-9822(01)00299-8 11470406

[B4] Baurain D. Brinkmann H. Petersen J. Rodríguez-Ezpeleta N. Stechmann A. Demoulin V.. (2010). Phylogenomic evidence for separate acquisition of plastids in cryptophytes, haptophytes, and stramenopiles. Mol. Biol. Evol. 27, 1698–1709. 10.1093/molbev/msq059 20194427

[B5] Baxter L. Tripathy S. Ishaque N. Boot N. Cabral A. Kemen E.. (2010). Signatures of adaptation to obligate biotrophy in the *Hyaloperonospora arabidopsidis* genome. Science 330, 1549–1551. 10.1126/science.1195203 21148394 PMC3971456

[B6] Bernstein E. Caudy A. A. Hammond S. M. Hannon G. J. (2001). Role for a bidentate ribonuclease in the initiation step of RNA interference. Nature 409, 363–366. 10.1038/35053110 11201747

[B7] Blanco F. A. Judelson H. S. (2005). A bZIP transcription factor from *Phytophthora* interacts with a protein kinase and is required for zoospore motility and plant infection. Mol. Microbiol. 56, 638–648. 10.1111/j.1365-2958.2005.04575.x 15819621

[B8] Braun L. Cannella D. Ortet P. Barakat M. Sautel C. F. Kieffer S.. (2010). A complex small RNA repertoire is generated by a plant/fungal-like machinery and effected by a metazoan-like Argonaute in the single-cell human parasite *Toxoplasma gondii*. PLoS Pathog. 6:e1000920. 10.1371/journal.ppat.1000920 20523899 PMC2877743

[B9] Burge C. Karlin S. (1997). Prediction of complete gene structures in human genomic DNA. J. Mol. Biol. 268, 78–94. 10.1006/jmbi.1997.0951 9149143

[B10] Cerutti H. Casas-Mollano J. A. (2006). On the origin and functions of RNA-mediated silencing: from protists to man. Curr. Genet. 50, 81–99. 10.1007/s00294-006-0078-x 16691418 PMC2583075

[B11] Dalmay T. Hamilton A. Rudd S. Angell S. Baulcombe D. C. (2000). An RNA-dependent RNA polymerase gene in *Arabidopsis* is required for posttranscriptional gene silencing mediated by a transgene but not by a virus. Cell 101, 543–553. 10.1016/S0092-8674(00)80864-8 10850496

[B12] de Jong D. Eitel M. Jakob W. Osigus H.-J. Hadrys H. Desalle R.. (2009). Multiple dicer genes in the early-diverging metazoa. Mol. Biol. Evol. 26, 1333–1340. 10.1093/molbev/msp042 19276153

[B13] Denli A. M. Hannon G. J. (2003). RNAi: an ever-growing puzzle. Trends Biochem. Sci. 28, 196–201. 10.1016/S0968-0004(03)00058-6 12713903

[B14] Derevnina L. Chin-Wo-Reyes S. Martin F. Wood K. Froenicke L. Spring O.. (2015). Genome sequence and architecture of the tobacco downy mildew pathogen, *Peronospora tabacina*. Mol. Plant Microbe Interact. 28, 1198–1215. 10.1094/MPMI-05-15-0112-R 26196322

[B15] Dlakić M. (2006). DUF283 domain of Dicer proteins has a double-stranded RNA-binding fold. Bioinformatics 22, 2711–2714. 10.1093/bioinformatics/btl468 16954143

[B16] Drinnenberg I. A. Weinberg D. E. Xie K. T. Mower J. P. Wolfe K. H. Fink G. R.. (2009). RNAi in budding yeast. Science 326, 544–550. 10.1126/science.1176945 19745116 PMC3786161

[B17] Dunoyer P. Himber C. Voinnet O. (2005). DICER-LIKE 4 is required for RNA interference and produces the 21-nucleotide small interfering RNA component of the plant cell-to-cell silencing signal. Nat. Genet. 37, 1356–1360. 10.1038/ng1675 16273107

[B18] Fahlgren N. Bollmann S. R. Kasschau K. D. Cuperus J. T. Press C. M. Sullivan C. M.. (2013). *Phytophthora* have distinct endogenous small RNA populations that include short interfering and microRNAs. PLoS ONE 8:e77181. 10.1371/journal.pone.0077181 24204767 PMC3804510

[B19] Fang Y. Tyler B. M. (2016). Efficient disruption and replacement of an effector gene in the oomycete *Phytophthora sojae* using CRISPR/Cas9. Mol. Plant Pathol. 17, 127–139. 10.1111/mpp.12318 26507366 PMC6638440

[B20] Finn R. D. Mistry J. Tate J. Coggill P. Heger A. Pollington J. E.. (2010). The Pfam protein families database. Nucleic Acids Res. 38, D211–D222. 10.1093/nar/gkp985 19920124 PMC2808889

[B21] Fry W. (2008). *Phytophthora infestans*: the plant (and R gene) destroyer. Mol. Plant Pathol. 9, 385–402. 10.1111/j.1364-3703.2007.00465.x 18705878 PMC6640234

[B22] Gasciolli V. Mallory A. C. Bartel D. P. Vaucheret H. (2005). Partially redundant functions of Arabidopsis DICER-like enzymes and a role for DCL4 in producing trans-acting siRNAs. Curr. Biol. 15, 1494–1500. 10.1016/j.cub.2005.07.024 16040244

[B23] Gaulin E. Jauneau A. Villalba F. Rickauer M. Esquerré-Tugayé M.-T. Bottin A. (2002). The CBEL glycoprotein of *Phytophthora parasitica var-nicotianae* is involved in cell wall deposition and adhesion to cellulosic substrates. J. Cell Sci. 115, 4565–4575. 10.1242/jcs.00138 12415001

[B24] Ghildiyal M. Zamore P. D. (2009). Small silencing RNAs: an expanding universe. Nat. Rev. 10, 94–108. 10.1038/nrg2504 19148191 PMC2724769

[B25] Grünwald N. J. Goss E. M. Press C. M. (2008). *Phytophthora ramorum*: a pathogen with a remarkably wide host range causing sudden oak death on oaks and ramorum blight on woody ornamentals. Mol. Plant Pathol. 9, 729–740. 10.1111/j.1364-3703.2008.00500.x 19019002 PMC6640315

[B26] Haas B. J. Kamoun S. Zody M. C. Jiang R. H. Y. Handsaker R. E. Cano L. M.. (2009). Genome sequence and analysis of the Irish potato famine pathogen *Phytophthora infestans*. Nature 461, 393–398. 10.1038/nature08358 19741609

[B27] Hamilton A. J. Baulcombe D. C. (1999). A species of small antisense RNA in posttranscriptional gene silencing in plants. Science 286, 950–952. 10.1126/science.286.5441.950 10542148

[B28] Hammond S. M. Bernstein E. Beach D. Hannon G. J. (2000). An RNA-directed nuclease mediates post-transcriptional gene silencing in *Drosophila* cells. Nature 404, 293–296. 10.1038/35005107 10749213

[B29] Hardham A. R. (2001). Investigations of oomycete cell biology, in Molecular and Cell Biology of Filamentous Fungi, ed Talbot N. (Oxford: Oxford University Press), 127–155.

[B30] Herr A. J. Jensen M. B. Dalmay T. Baulcombe D. C. (2005). RNA polymerase IV directs silencing of endogenous DNA. Science 308, 118–120. 10.1126/science.1106910 15692015

[B31] Hinas A. Reimegård J. Wagner E. G. H. Nellen W. Ambros V. R. Söderbom F. (2007). The small RNA repertoire of *Dictyostelium discoideum* and its regulation by components of the RNAi pathway. Nucleic Acids Res. 35, 6714–6726. 10.1093/nar/gkm707 17916577 PMC2175303

[B32] Huang A. He L. Wang G. (2011). Identification and characterization of microRNAs from *Phaeodactylum tricornutum* by high-throughput sequencing and bioinformatics analysis. BMC Genomics 12:337. 10.1186/1471-2164-12-337 21718527 PMC3141676

[B33] Huelsenbeck J. P. Ronquist F. (2001). MRBAYES: Bayesian inference of phylogenetic trees. Bioinformatics 17, 754–755. 10.1093/bioinformatics/17.8.754 11524383

[B34] Jiang R. H. Y. de Bruijn I. Haas B. J. Belmonte R. Löbach L. Christie J.. (2013). Distinctive expansion of potential virulence genes in the genome of the oomycete fish pathogen *Saprolegnia parasitica*. PLoS Genet. 9:e1003272. 10.1371/journal.pgen.1003272 23785293 PMC3681718

[B35] Judelson H. S. Tani S. (2007). Transgene-induced silencing of the zoosporogenesis-specific NIFC gene cluster of *Phytophthora infestans* involves chromatin alterations. Eukaryotic Cell 6, 1200–1209. 10.1128/EC.00311-06 17483289 PMC1951104

[B36] Judelson H. S. Whittaker S. L. (1995). Inactivation of transgenes in *Phytophthora infestans* is not associated with their deletion, methylation, or mutation. Curr. Genet. 28, 571–579. 10.1007/BF00518171 8593689

[B37] Kamoun S. (2003). Molecular genetics of pathogenic oomycetes. Eukaryotic Cell 2, 191–199. 10.1128/EC.2.2.191-199.2003 12684368 PMC154851

[B38] Kasschau K. D. Fahlgren N. Chapman E. J. Sullivan C. M. Cumbie J. S. Givan S. A.. (2007). Genome-wide profiling and analysis of *Arabidopsis* siRNAs. PLoS Biol. 5:e57. 10.1371/journal.pbio.0050057 17298187 PMC1820830

[B39] Kearse M. Moir R. Wilson A. Stones-Havas S. Cheung M. Sturrock S.. (2012). Geneious basic: an integrated and extendable desktop software platform for the organization and analysis of sequence data. Bioinformatics 28, 1647–1649. 10.1093/bioinformatics/bts199 22543367 PMC3371832

[B40] Kuhlmann M. Borisova B. E. Kaller M. Larsson P. Stach D. Na J.. (2005). Silencing of retrotransposons in Dictyostelium by DNA methylation and RNAi. Nucleic Acids Res. 33, 6405–6417. 10.1093/nar/gki952 16282589 PMC1283529

[B41] Latijnhouwers M. Govers F. (2003). A *Phytophthora infestans* G-protein beta subunit is involved in sporangium formation. Eukaryotic Cell 2, 971–977. 10.1128/EC.2.5.971-977.2003 14555479 PMC219352

[B42] Lee H.-C. Li L. Gu W. Xue Z. Crosthwaite S. K. Pertsemlidis A.. (2010). Diverse pathways generate microRNA-like RNAs and Dicer-independent small interfering RNAs in fungi. Mol. Cell 38, 803–814. 10.1016/j.molcel.2010.04.005 20417140 PMC2902691

[B43] Lee R. C. Feinbaum R. L. Ambros V. (1993). The *C. elegans* heterochronic gene lin-4 encodes small RNAs with antisense complementarity to lin-14. Cell 75, 843–854. 10.1016/0092-8674(93)90529-Y 8252621

[B44] Lee S. R. Collins K. (2007). Physical and functional coupling of RNA-dependent RNA polymerase and Dicer in the biogenesis of endogenous siRNAs. Nat. Struct. Mol. Biol. 14, 604–610. 10.1038/nsmb1262 17603500

[B45] Lee Y. Ahn C. Han J. Choi H. Kim J. Yim J.. (2003). The nuclear RNase III Drosha initiates microRNA processing. Nature 425, 415–419. 10.1038/nature01957 14508493

[B46] Lepère G. Nowacki M. Serrano V. Gout J.-F. Guglielmi G. Duharcourt S.. (2009). Silencing-associated and meiosis-specific small RNA pathways in *Paramecium tetraurelia*. Nucleic Acids Res. 37, 903–915. 10.1093/nar/gkn1018 19103667 PMC2647294

[B47] Lévesque C. A. Brouwer H. Cano L. Hamilton J. P. Holt C. Huitema E.. (2010). Genome sequence of the necrotrophic plant pathogen *Pythium ultimum* reveals original pathogenicity mechanisms and effector repertoire. Genome Biol. 11:R73. 10.1186/gb-2010-11-7-r73 20626842 PMC2926784

[B48] Links M. G. Holub E. Jiang R. H. Y. Sharpe A. G. Hegedus D. Beynon E.. (2011). De novo sequence assembly of *Albugo candida* reveals a small genome relative to other biotrophic oomycetes. BMC Genomics 12:503. 10.1186/1471-2164-12-503 21995639 PMC3206522

[B49] Liu Q. Feng Y. Zhu Z. (2009). Dicer-like (DCL) proteins in plants. Funct. Integr. Genomics 9, 277–286. 10.1007/s10142-009-0111-5 19221817

[B50] MacRae I. J. Zhou K. Li F. Repic A. Brooks A. N. Cande W. Z.. (2006). Structural basis for double-stranded RNA processing by Dicer. Science 311, 195–198. 10.1126/science.1121638 16410517

[B51] Makeyev E. V. Bamford D. H. (2002). Cellular RNA-dependent RNA polymerase involved in posttranscriptional gene silencing has two distinct activity modes. Mol. Cell 10, 1417–1427. 10.1016/S1097-2765(02)00780-3 12504016

[B52] Marker S. Le Mouël A. Meyer E. Simon M. (2010). Distinct RNA-dependent RNA polymerases are required for RNAi triggered by double-stranded RNA versus truncated transgenes in *Paramecium tetraurelia*. Nucleic Acids Res. 38, 4092–4107. 10.1093/nar/gkq131 20200046 PMC2896523

[B53] Martens H. Novotny J. Oberstrass J. Steck T. L. Postlethwait P. Nellen W. (2002). RNAi in *Dictyostelium*: the role of RNA-directed RNA polymerases and double-stranded RNase. Mol. Biol. Cell 13, 445–453. 10.1091/mbc.01-04-0211 11854403 PMC65640

[B54] Mehta A. Mann M. Zhao J. L. Marinov G. K. Majumdar D. Garcia-Flores Y.. (2015). The microRNA-212/132 cluster regulates B cell development by targeting Sox4. J. Exp. Med. 212, 1679–1692. 10.1083/jcb.2107OIA191 26371188 PMC4577845

[B55] Nakai K. Horton P. (1999). PSORT: a program for detecting sorting signals in proteins and predicting their subcellular localization. Trends Biochem. Sci. 24, 34–36. 10.1016/S0968-0004(98)01336-X 10087920

[B56] Norden-Krichmar T. M. Allen A. E. Gaasterland T. Hildebrand M. (2011). Characterization of the small RNA transcriptome of the diatom, *Thalassiosira pseudonana*. PLoS ONE 6:e22870. 10.1371/journal.pone.0022870 21857960 PMC3155517

[B57] Park W. Li J. Song R. Messing J. Chen X. (2002). CARPEL FACTORY, a Dicer homolog, and HEN1, a novel protein, act in microRNA metabolism in *Arabidopsis thaliana*. Curr. Biol. 12, 1484–1495. 10.1016/S0960-9822(02)01017-5 12225663 PMC5137372

[B58] Pfaffl M. W. Horgan G. W. Dempfle L. (2002). Relative expression software tool (REST) for group-wise comparison and statistical analysis of relative expression results in real-time PCR. Nucleic Acids Res. 30:e36. 10.1093/nar/30.9.e36 11972351 PMC113859

[B59] Pfaffl M. W. Tichopad A. Prgomet C. Neuvians T. P. (2004). Determination of stable housekeeping genes, differentially regulated target genes and sample integrity: bestkeeper–excel-based tool using pair-wise correlations. Biotechnol. Lett. 26, 509–515. 10.1023/B:BILE.0000019559.84305.47 15127793

[B60] Qutob D. Hraber P. T. Sobral B. W. Gijzen M. (2000). Comparative analysis of expressed sequences in *Phytophthora sojae*. Plant Physiol. 123, 243–254. 10.1104/pp.123.1.243 10806241 PMC58998

[B61] Reinhart B. J. Weinstein E. G. Rhoades M. W. Bartel B. Bartel D. P. (2002). MicroRNAs in plants. Genes Dev. 16, 1616–1626. 10.1101/gad.1004402 12101121 PMC186362

[B62] Ronquist F. Huelsenbeck J. P. (2003). MrBayes 3: Bayesian phylogenetic inference under mixed models. Bioinformatics 19, 1572–1574. 10.1093/bioinformatics/btg180 12912839

[B63] Rozen S. Skaletsky H. (2000). Primer3 on the WWW for general users and for biologist programmers. Methods Mol. Biol. 132, 365–386. 10.1385/1-59259-192-2:365 10547847

[B64] Schauer S. E. Jacobsen S. E. Meinke D. W. Ray A. (2002). DICER-LIKE1: blind men and elephants in Arabidopsis development. Trends Plant Sci. 7, 487–491. 10.1016/S1360-1385(02)02355-5 12417148

[B65] Shi H. Tschudi C. Ullu E. (2006). An unusual Dicer-like1 protein fuels the RNA interference pathway in *Trypanosoma brucei*. RNA 12, 2063–2072. 10.1261/rna.246906 17053086 PMC1664728

[B66] Soifer H. S. Sano M. Sakurai K. Chomchan P. Saetrom P. Sherman M. A.. (2008). A role for the Dicer helicase domain in the processing of thermodynamically unstable hairpin RNAs. Nucleic Acids Res. 36, 6511–6522. 10.1093/nar/gkn687 18927112 PMC2582626

[B67] Tabara H. Sarkissian M. Kelly W. G. Fleenor J. Grishok A. Timmons L.. (1999). The rde-1 gene, RNA interference, and transposon silencing in C. elegans. Cell 99, 123–132. 10.1016/S0092-8674(00)81644-X 10535731

[B68] Tahbaz N. Kolb F. A. Zhang H. Jaronczyk K. Filipowicz W. Hobman T. C. (2004). Characterization of the interactions between mammalian PAZ PIWI domain proteins and Dicer. EMBO Rep. 5, 189–194. 10.1038/sj.embor.7400070 14749716 PMC1298981

[B69] Tyler B. M. (2007). *Phytophthora sojae*: root rot pathogen of soybean and model oomycete. Mol. Plant Pathol. 8, 1–8. 10.1111/j.1364-3703.2006.00373.x 20507474

[B70] Tyler B. M. Tripathy S. Zhang X. Dehal P. Jiang R. H. Y. Aerts A.. (2006). *Phytophthora* genome sequences uncover evolutionary origins and mechanisms of pathogenesis. Science 313, 1261–1266. 10.1126/science.1128796 16946064

[B71] Tyler B. M. Wu M. Wang J. Cheung W. Morris P. F. (1996). Chemotactic preferences and strain variation in the response of *Phytophthora sojae* zoospores to host isoflavones. Appl. Environ. Microbiol. 62, 2811–2817. 16535375 10.1128/aem.62.8.2811-2817.1996 PMC1388913

[B72] Vagin V. V. Sigova A. Li C. Seitz H. Gvozdev V. Zamore P. D. (2006). A distinct small RNA pathway silences selfish genetic elements in the germline. Science 313, 320–324. 10.1126/science.1129333 16809489

[B73] van West P. Kamoun S. van't Klooster J. W. Govers F. (1999). Internuclear gene silencing in *Phytophthora infestans*. Mol. Cell 3, 339–348. 10198636 10.1016/s1097-2765(00)80461-x

[B74] Vetukuri R. R. Åsman A. K. M. Tellgren-Roth C. Jahan S. N. Reimegård J. Fogelqvist J.. (2012). Evidence for small RNAs homologous to effector-encoding genes and transposable elements in the oomycete *Phytophthora infestans*. PLoS ONE 7:e51399. 10.1371/journal.pone.0051399 23272103 PMC3522703

[B75] Vetukuri R. R. Avrova A. O. Grenville-Briggs L. J. Van West P. Söderbom F. Savenkov E. I.. (2011). Evidence for involvement of Dicer-like, Argonaute and histone deacetylase proteins in gene silencing in *Phytophthora infestans*. Mol. Plant Pathol. 12, 772–785. 10.1111/j.1364-3703.2011.00710.x 21726377 PMC6640358

[B76] Wightman B. Ha I. Ruvkun G. (1993). Posttranscriptional regulation of the heterochronic gene lin-14 by lin-4 mediates temporal pattern formation in C. elegans. Cell 75, 855–862. 10.1016/0092-8674(93)90530-4 8252622

[B77] Xie Z. Allen E. Wilken A. Carrington J. C. (2005). DICER-LIKE 4 functions in trans-acting small interfering RNA biogenesis and vegetative phase change in *Arabidopsis thaliana*. Proc. Natl. Acad. Sci. U.S.A. 102, 12984–12989. 10.1073/pnas.0506426102 16129836 PMC1200315

[B78] Xie Z. Johansen L. K. Gustafson A. M. Kasschau K. D. Lellis A. D. Zilberman D.. (2004). Genetic and functional diversification of small RNA pathways in plants. PLoS Biol. 2:E104. 10.1371/journal.pbio.0020104 15024409 PMC350667

[B79] Yu D. Fan B. MacFarlane S. A. Chen Z. (2003). Analysis of the involvement of an inducible *Arabidopsis* RNA-dependent RNA polymerase in antiviral defense. Mol. Plant Microbe Interact. 16, 206–216. 10.1094/MPMI.2003.16.3.206 12650452

[B80] Zamore P. D. Tuschl T. Sharp P. A. Bartel D. P. (2000). RNAi: double-stranded RNA directs the ATP-dependent cleavage of mRNA at 21 to 23 nucleotide intervals. Cell 101, 25–33. 10.1016/S0092-8674(00)80620-0 10778853

[B81] Zong J. Yao X. Yin J. Zhang D. Ma H. (2009). Evolution of the RNA-dependent RNA polymerase (RdRP) genes: duplications and possible losses before and after the divergence of major eukaryotic groups. Gene 447, 29–39. 10.1016/j.gene.2009.07.004 19616606

