# Supplementary material for: Diverse Evolutionary Trajectories for Small RNA Biogenesis Genes in the Oomycete Genus Phytophthora
Source: Front Plant Sci. 2016 Mar 15;7:284. doi: 10.3389/fpls.2016.00284 (PMC4791657; doi:10.3389/fpls.2016.00284)
Supplement: Supplementary file 1 [file DataSheet1.DOCX]

Supplementary Material

**Diverse evolutionary trajectories for small RNA biogenesis genes in the oomycete genus *Phytophthora***

**Stephanie R. Bollmann, Yufeng Fang, Caroline M. Press, Brett M. Tyler, and Niklaus J. Grünwald^*^**

^*^**Correspondence:** Niklaus J. Grünwald: [nik.grunwald@ars.usda.gov](mailto:grunwaln@science.oregonstate.edu)

**1 Supplementary Data**

Data files 1-4 provide the fasta alignments used in the phylogenetic analyses, data file 5 provides the full amino acid sequences of the DCR and RDR homologs used for the phylogenetic analyses.

**2 Supplementary Figures**

**Supplementary Figure 1.** Primers for cloning *P. sojae* small RNA biogenesis components (DCL and RDR). Genes are labeled followed by their Gene ID from the Eumicrobe Database. In the genomic DNA diagrams, exons and introns are represented as black bars and lines, respectively. 5′ and 3′ UTRs are represented as grey bars. (A-C). Positions of primers used for cloning DCL1, DCL2, and RDR from cDNA, as detailed in Supplementary Table 1.

**Supplementary Figure 2.** Gene structure consensus sequence comparisons of DCL1, DCL2, and RDR. Transcription start site (A), translation start site (B), and exon/intron boundary (C) sequences for DCL1, DCL2, and RDR are compared against the *Phytophthora* consensus sequence as described by Kamoun (2003). Underlined bases indicate: (A) first base of cDNA, (B) translation start codon, and (C) first and last bases of intron.

**Supplementary Figure 3.** Mean relative expression of DCL and RDR homologs. Mean expression of DCL1, DCL2 and RDR relative to mycelium and normalized by reference genes WS41 and β-Tubulin. RT-qPCR data is based on 6 biological replicates. MYC: mycelium; ZOO: zoospores; GC: germinated cysts. Labels a/b/c across individual genes indicate significantly different means based on Tukey’s test (p < 0.05; shown are means and standard deviations).

**Supplementary Figure 4.** Additional representative images of DCL subcellular localization in *P. sojae*. (A) Subcellular localization of PsDcl1; (B) Subcellular localization of PsDcl2.

**Supplementary Figure 5.** Consensus tree of Dicer homologs based on RNaseIIIa and RNaseIIIb domains individually. (A) Radial tree. Significance of Bayesian support is indicated as thickness of branches. (B) Rectangular tree. Bayesian support values are shown next to nodes. (A,B) Branches are colored to denote major species groups, as in Figures 2, 3, 4 and 5. Species abbreviations are defined in Supplementary Table 2A.

**Supplementary Figure 6.** Catalytic residues for RNase III domain. Alignment of RNase III domains from phylogenetic analysis. Red boxes indicate the first and second loci with catalytic activity, which show strong conservation. Orange ovals indicate deviation from the consensus. Species abbreviations are defined in Supplementary Table 2A.

**Supplementary Figure 7.** Catalytic residues for RDRP domain. Alignment of RDRP domains from phylogenetic analysis. Residues 220-300 from the RDRP domain are shown. Red boxes indicate the locus with catalytic activity, which shows strong conservation. Orange ovals indicate deviation from the consensus (more than one change in complete sequences). Changes in the second residue of the consensus sequence were not highlighted if that was the only change seen. Species abbreviations are defined in Supplementary Table 2B.

**Supplementary Figure 8.** Catalytic residues for DEAD-box domain. Alignment of DEAD-box domains from phylogenetic analysis. Red boxes indicate the locus with catalytic activity, which shows strong conservation. Orange ovals indicate deviation from the consensus. Species abbreviations are defined in Supplementary Table 2A.

**Supplementary Figure 9.** Conservation of key residues in the DEAD-box helicase domain. Amino acid sequences of the Oomycete DCL1 and RDR homologs at the ATP-binding and Mg^2+^-binding sites.

**3 Supplementary Tables**

Supplementary table 1 lists the primers used for cloning DCR and RDR homologs, and supplementary tables 2A and 2B list taxonomic information for the species used in the phylogenetic analyses.
