# Supplementary material for: Diverse Evolutionary Trajectories for Small RNA Biogenesis Genes in the Oomycete Genus Phytophthora
Source: Front Plant Sci. 2016 Mar 15;7:284. doi: 10.3389/fpls.2016.00284 (PMC4791657; doi:10.3389/fpls.2016.00284)
Supplement: Supplementary file 2 [file DataSheet2.PDF]

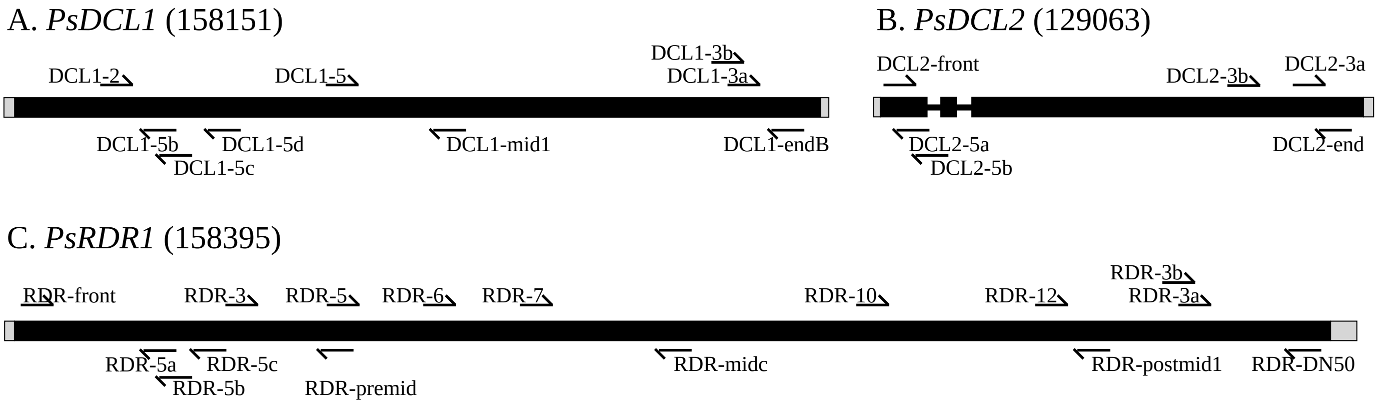

Supplementary Figure 1. Primers for cloning P. sojae small RNA biogenesis components (DCL and RDR). Genes are labeled followed by their Gene ID from the Eumicrobe Database. In the genomic DNA diagrams, exons and introns are represented as black bars and lines, respectively. 5′ and 3′ UTRs are represented as grey bars. (A-C). Positions of primers used for cloning DCL1, DCL2, and RDR from cDNA, as detailed in Supplementary Table 1.

A. Transcription start site

GCTCATTYBNNNWTTY – consensus

TGTCACTTGCATTTTT – DCL1 52b 5′ UTR (major)

CATCATTTCCGCAATT – DCL1 18b 5′ UTR (minor)

TCTCATTCCGTGATTT – DCL2 33b 5′ UTR

TCTCACTCCCAGATTT – RDR 32b 5′ UTR

B. Translation start site

ACCATGA – consensus

GCCATGG – DCL1

GTGATGG – DCL2

GCGATGA – RDR

C. Exon/intron boundaries

GTRNGT…YAG – consensus

GTAAGC…AAG – DCL2 intron 1 (71b)

GTGAGC…CAG – DCL2 intron 2 (83b)

Supplementary Figure 2. Gene structure consensus sequence comparisons of DCL1, DCL2, and RDR. Transcription start site (A), translation start site (B), and exon/intron boundary (C) sequences for DCL1, DCL2, and RDR are compared against the Phytophthora consensus sequence as described by Kamoun (2003). Underlined bases indicate: (A) first base of cDNA, (B) translation start codon, and (C) first and last bases of intron.

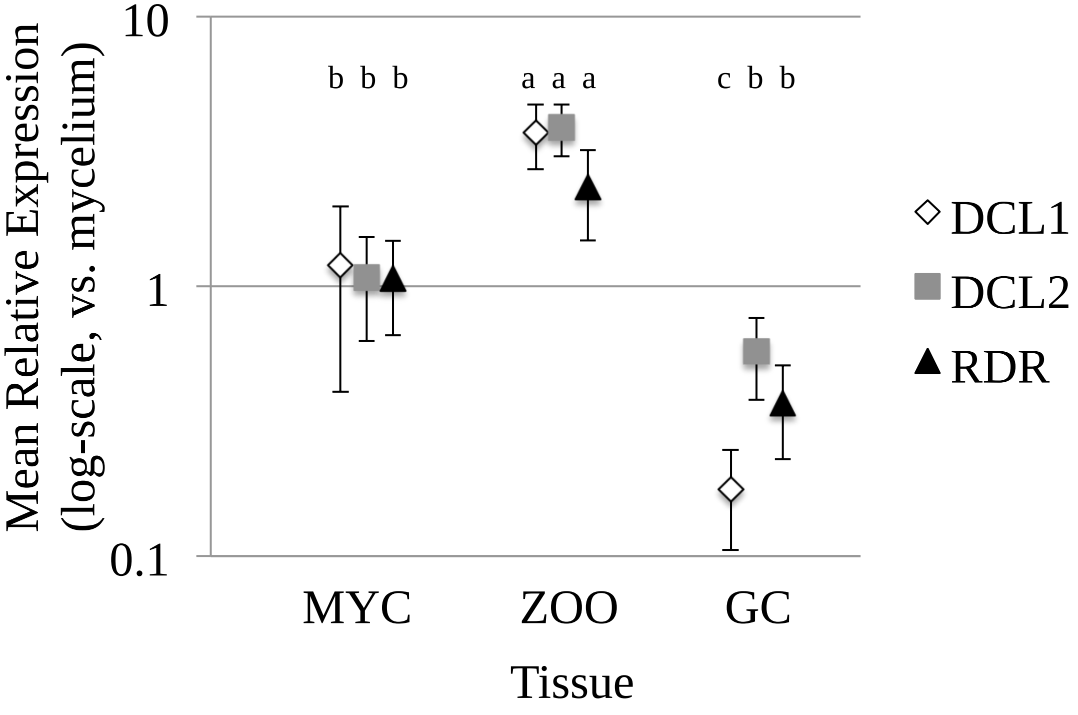

Supplementary Figure 3. Mean relative expression of DCL and RDR homologs. Mean expression of DCL1, DCL2 and RDR relative to mycelium and normalized by reference genes WS41 and β-Tubulin. RT-qPCR data is based on 6 biological replicates. MYC: mycelium; ZOO: zoospores; GC: germinated cysts. Labels a/b/c across individual genes indicate significantly different means based on Tukey's test ($p < 0.05$; shown are means and standard deviations).

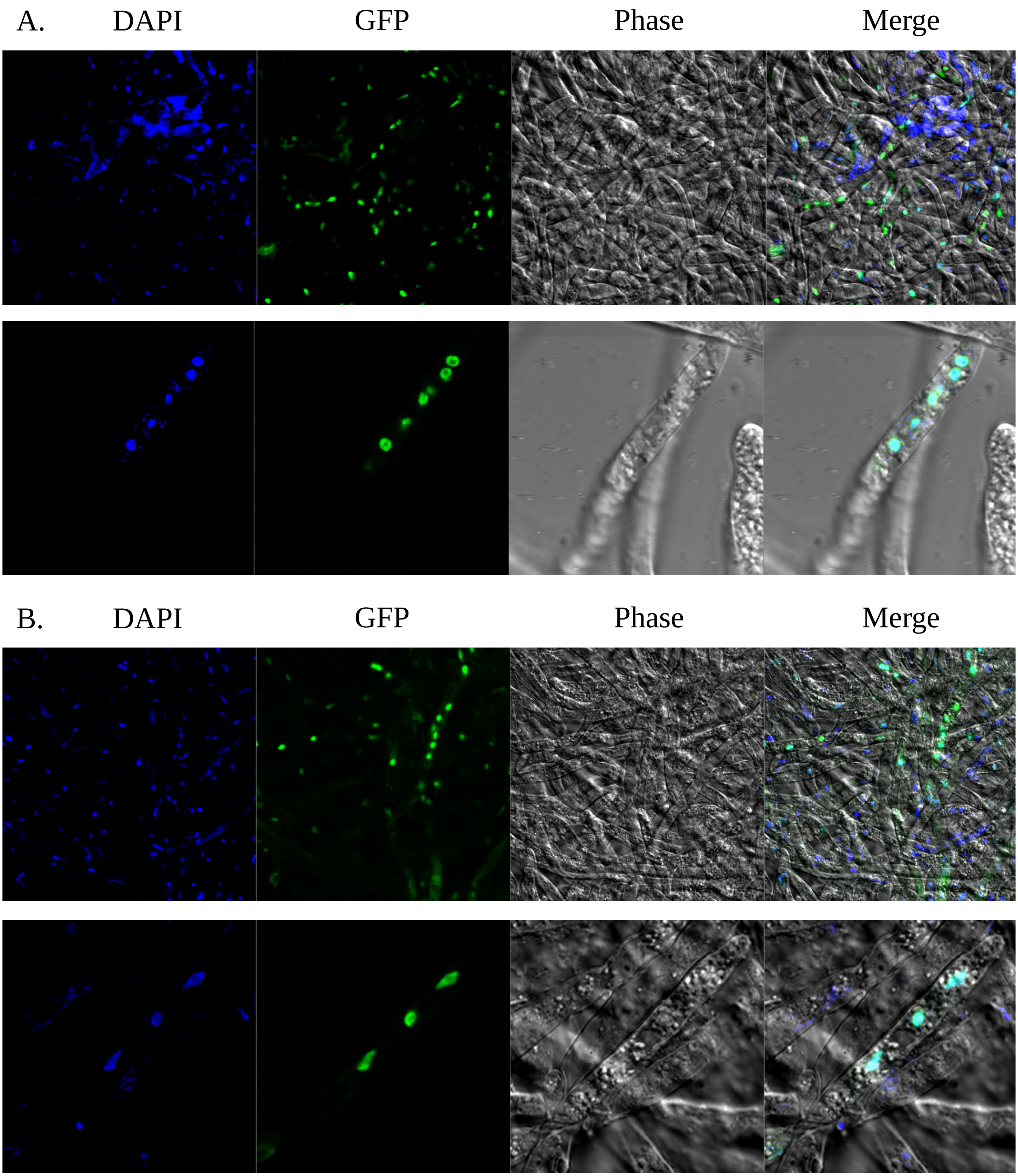

Supplementary Figure 4. Additional representative images of DCL subcellular localization in P. sojae.
(A) Subcellular localization of PsDcl1; (B) Subcellular localization of PsDcl2.

Supplementary Figure 5. Consensus tree of Dicer homologs based on RNaseIIIa and RNaseIIIb domains individually. (A) Radial tree. Significance of Bayesian support is indicated as thickness of branches. (B) Rectangular tree. Bayesian support values are shown next to nodes. (A,B) Branches are colored to denote major species groups, as in Figures 2, 3, 4 and 5. Species abbreviations are defined in Supplementary Table 2A.

Supplementary Figure 5A

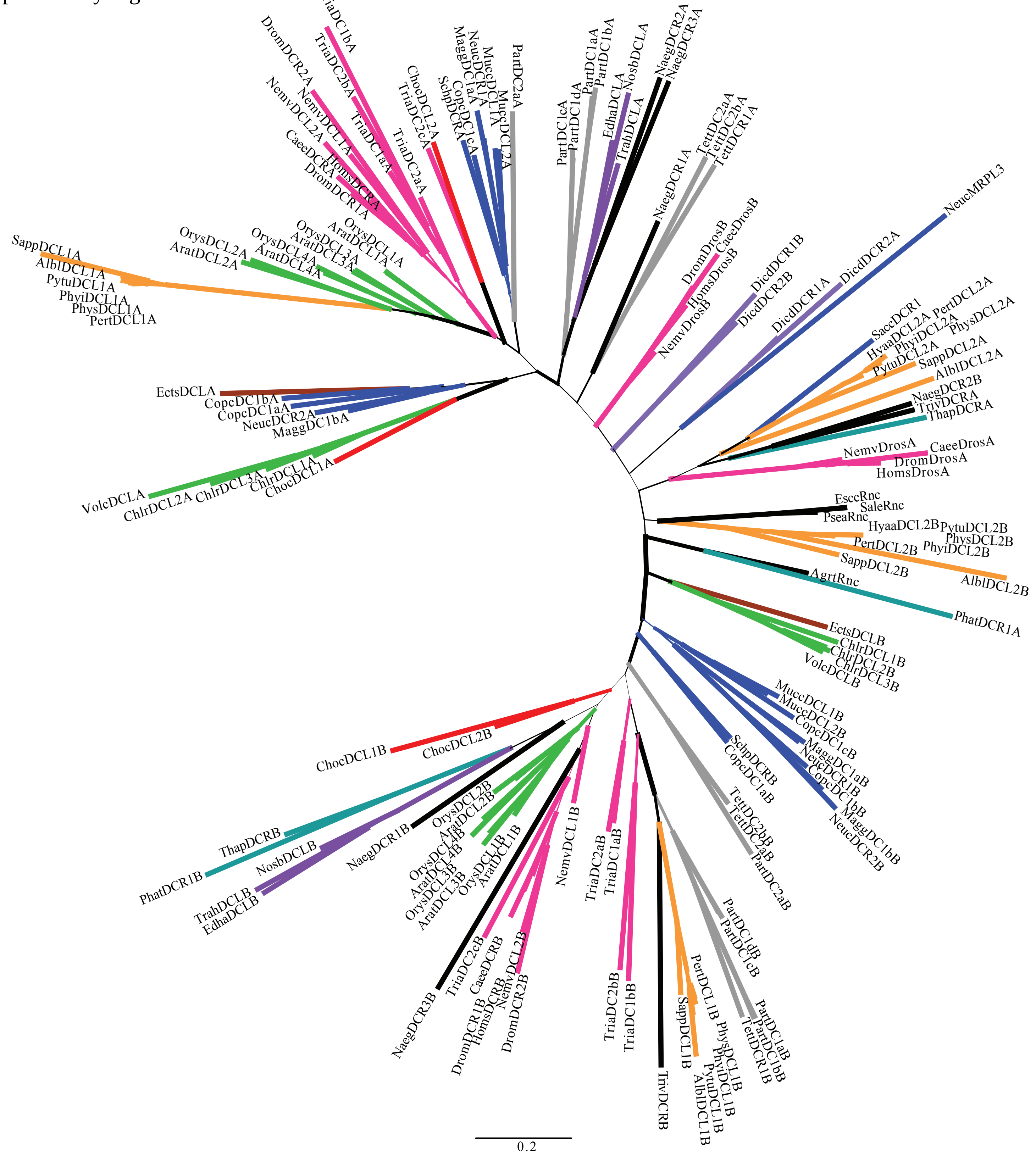

Supplementary Figure 5B

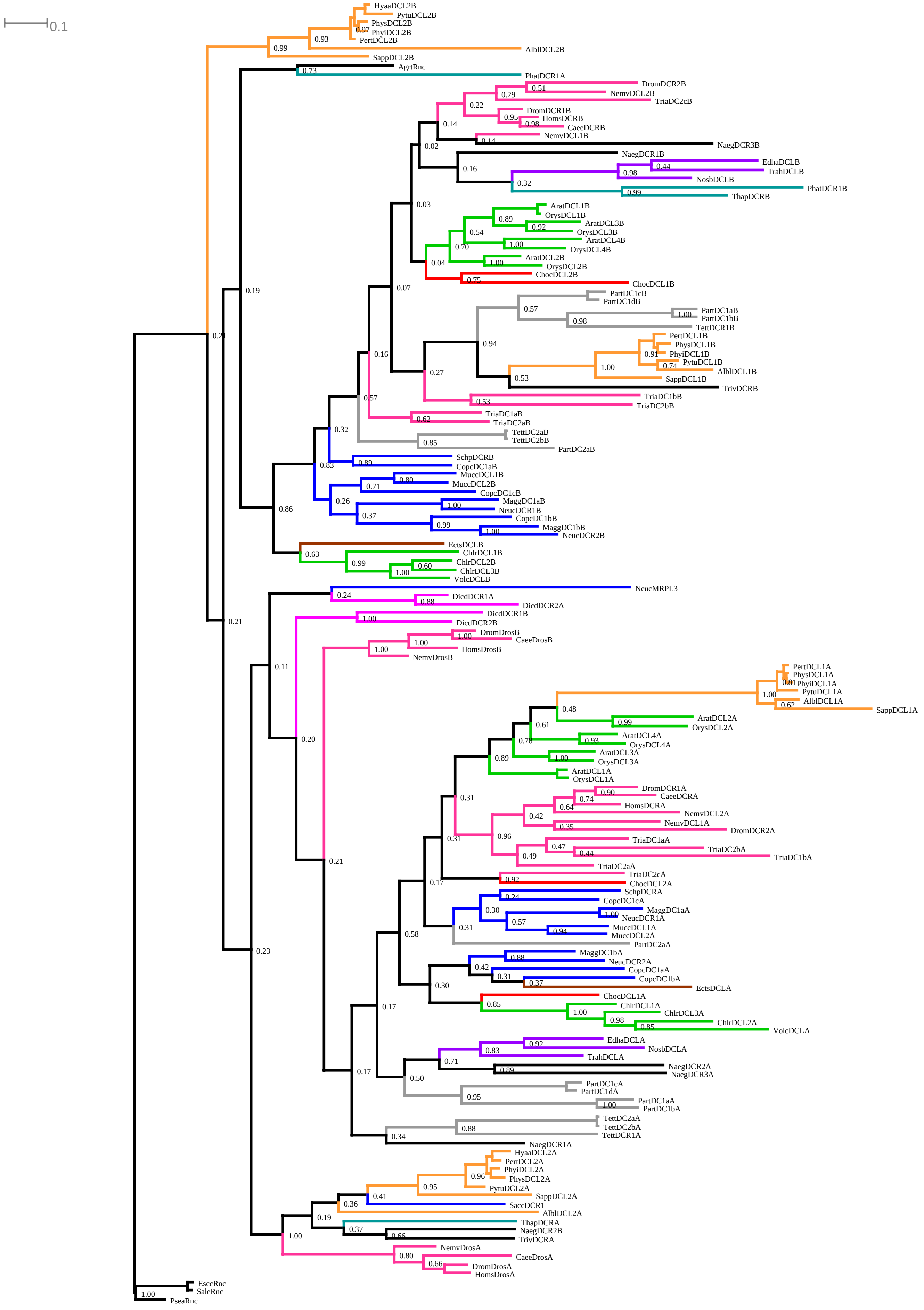

Supplementary Figure 6. Catalytic residues for RNase III domain. Alignment of RNase III domains from phylogenetic analysis. Red boxes indicate the first and second loci with catalytic activity, which show strong conservation. Orange ovals indicate deviation from the consensus. Species abbreviations are defined in Supplementary Table 2A.

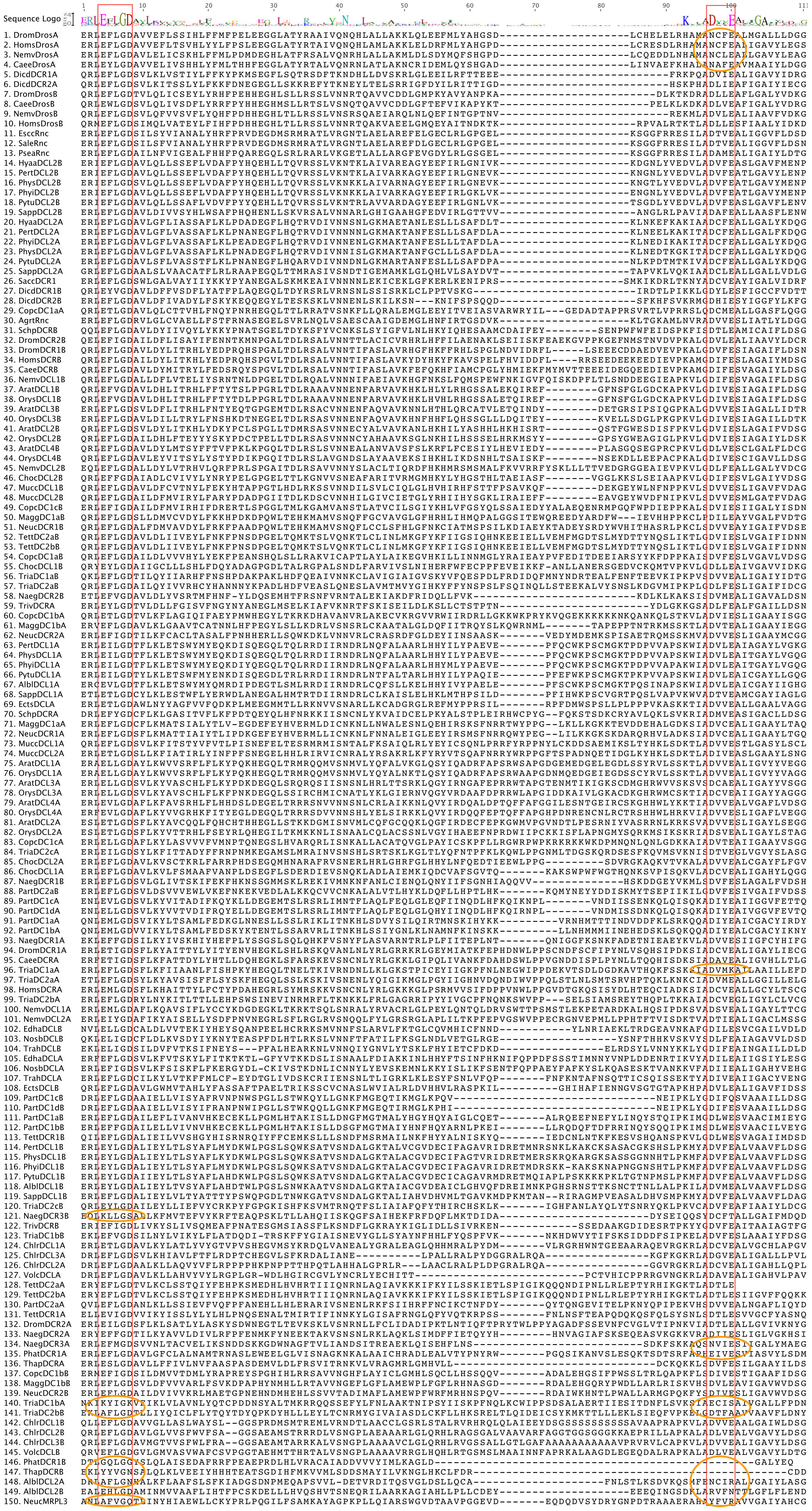

Supplementary Figure 7. Catalytic residues for RDRP domain. Alignment of RDRP domains from phylogenetic analysis. Residues 220-300 from the RDRP domain are shown. Red boxes indicate the locus with catalytic activity, which shows strong conservation. Orange ovals indicate deviation from the consensus (more than one change in complete sequences). Changes in the second residue of the consensus sequence were not highlighted if that was the only change seen. Species abbreviations are defined in Supplementary Table 2B.

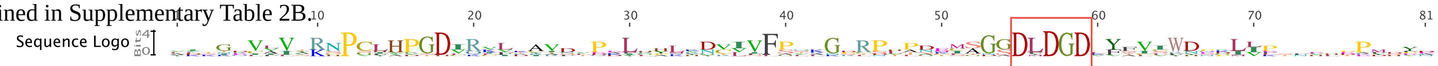


```
1. AratRDR1      IITGPVVVAKNPCLHPGDVRVLQAVNVPALNHMVDCVVFPQKGLRPHPNECSGSDLDGDIYFVCWDQELVPPRTSEPMDYT
2. OrysRDR1      TVTGKVVIAKNPCLHPGDIRILHAVDVPVLHHMFNCVVFPQQGPRPHPNECSGSDLDGDIYFVSWDPSLIPPRMVTPMDYT
3. AratRDR2      VVIGKVVVTKNPCLHPGDIRVLDAIYEVHFKGYLDCIIFPQKGERPHPNECSGGDLDGDQFFVSWDEKIIPSEMDPPMDYA
4. OrysRDR2      TVVGKVAITKNPCLHPGDIRVLEAIYDPDLVGMVDCLVFPQRGERPHPNECSGGDLDGDLYFITWDDKLIPEKVDTPMDYT
5. AratRDR6      VVKGYVAIAKNPCLHPGDVRILEAVDVPQLHHMYDCLIFPQKGDRPHTNEASGSDLDGDLYFVAWDQKLIPPKSYPAMHYD
6. OrysRDR6      VILGTVVIAKNPCLHPGDVRILEAVDVPELHHLVDCLVFPQKGERPHANEASGSDLDGDLYFVTWDEKLIPPKSWNPMDYS
7. MuccRDR1      LITGDVVVFRNPCFHPGDVRIVKAVDREELHYLVDVIVFSSKGVRDIPSMCSGGDLDGDDYTVYWDPRLIPKGNYEPMDYT
8. NeucRDR2      VITGTCVVGRNPSLHPGDIRVVEAVDVPALRHLRDVVVFPLTGDRDVPSMCSGGDLDGDDFFVIWDPLLIPKRSHPPMISE
9. SchpRDR       VIVGLCIVARNPSLHPGDVRVCKAVRCDELMHLKNVIVFPTTGDRSIPAMCSGGDLDGDEYTVIWDQRLLPKVNYPPLLES
10. IxosRDR1a    ILKGDVIVTKNPCMHPGDIRRLTAVDVPRLHHVRDCIVFPAKGERPHPDEMSGSDLDGDEYSVLWYEDLIFRNNCSPMHYY
11. IxosRDR1b    LRAGRVMVTKNPCIHPGDIRLLEAVDVPALSHIRDCVVFPQIGKRPHPDEMAGSDLDGDEYSVIWYEPLLFKRNDDPMDFY
12. IxosRDR2a    VITGQVLVTKCPCLHPGDVRKFTAIDEPRLHHVVDCIVFPGQGHRPHPDEMAGSDLDGDEYIVIWEPSLLFPYNKQPMNFS
13. IxosRDR2b    FCTGAVLVTKCPCLHPGDVRKFTAVDVPALHHVKDCVVFPARGPRPHPNEMAGSDLDGDEYVVIWKKDLLFPPNRKAMVFS
14. NemvRDR1     VLKGPVVVTRNPCLHPGDVRRLVAIDTPKLRHLVDCVVFPKTGQRPHPSEMAGGDLDGDLYFVCWYKPLIPSQEIDPMDYK
15. NemvRDR6     ILTGDVVVSKNPCFHPGDMRKFNAVDIPALYHLVDCIVFPAKGPRPHTDEMSGSDLDGDKYFATWLRGIIPDDNEPPMDFY
16. CocsRDR      IVTGTVVVAKNPCFHPGDVRKFQAFRSPELHHLVNCLVFPQKGQRPHPDECSGSDLDGDQYFVSWEPGLLFPPNREPMSFH
17. NaegRDR1c    VVKGRCIVGKNPSLHKGDLRIVTAVDRPELYHLNDVVVFSKKGKRPITNMITGSDLDGDQFFVYWDPDLIPESDSEPMDYS
18. SappRDR1     AFGSSVAVGRCPCLHPGDIQCLTLVDAPALRHLYDVLVFSSAGDRPDPDTMAGGDLDGDVYFCIWDPRLLPTTVYPPMEAA
19. SapdRDR1     AFGSTIAVGRCPCLHPGDIQCLTLVDAPALRHLYDVLVFSSAGDRPDPDTMAGGDLDGDVYFCIWDERLLPTTTYPPMEPG
20. SappRDR4     PPNAIIAVGRCPCLHPGDIRRLTYVDVPALHHLHDVVVFSSHGDRPTPDTMAGGDLDGDVYFCIWDAHLVPHDEVAPMTRP
21. SapdRDR2     PPHATLAVGRCPCLHPGDIRRLTYVHVPALSHLHDVVVFSAHGDRPTPDTMAGGDLDGDIYFCIWNQKLVPPTEFAPMTKP
22. SappRDR2     ALGTRCVVGRNPSLHPGDLRILTYADVPALHHLYDVLVFSSRGDRPVTDMMSGGDLDGDLYFCLWDHRLVPAQDVPPMHPP
23. SappRDR3     ALGTRCVVGRNPSLHPGDLRILTYTDVPALHHLYDVLVFSSRGDRPVTDMMSGGDLDGDLYFCLWDHRLVPAQDVPPMPPP
24. SapdRDR3     AFGTRCVVGRNPSLHPGDLRVLTYTNAPALRHLFDVIVFSSRGQRP
25. SappRDR5     ALGTRCVVGRNPSLHPGDLRILTYADVPALHHLYDVLVFSSRGDRPVTDMMSGGDLDGDLYFCLWDHRLVPAQDVPPMHPP
26. SapdRDR4     TIGTRCVVGRHPSLHPGDLRVLTYMMVPALCHLFDVLVFSSRGQRPVTDMMSGGDMDGDLYFCLWDQRLVPAQDVPPMQPP
27. CaeeRDR1     LKTGKVLITKNPCHVPGDVRVFDAVWQPALAHLVDVVVFPQHGPRPHPDEMAGSDLDGDEYSIIWDQEMLLDYNEEAMVFP
28. CaeeRDR2     VLTGTVLLTKNPCIVAGDVRIFEAVDIPELHHMCDVVVFPQHGPRPHPDEMAGSDLDGDEYSIIWDQQLLLDKNEDPYDFT
29. CaeeRDR6a    IVTGTVLLTKNPCIVPGDVRIFEAVDIPELHHMCDVVVFPQHGPRPHPDEMAGSDLDGDEYSVIWDQELLLERNEEPFDFA
30. CaeeRDR6b    IVTGTVLLTKNPGIVPGDVRIFEAVDIPELHHLCDVVVFPQHGPRPHPDEMAGSDLDGDEYSVIWDQKLLLERNEEAFDFA
31. HyaaRDR      LDNVDVVVHRSPCHHPGDIRVLYRIDVPPLRQLKDCIVFPCEGPRPHPDECTGGDLDGDTFVVIWDERLIPSRTKEPMTFD
32. PertRDR      LDGIDVAVHRSPCHHPGDIRVLRRNDVPQLRQLKDCIVFPCQGPRPHPDECTGGDLDGDIFVVIWDKRLIPSRTKEPMVFD
33. PhyiRDR      LDGIDVAVHRSPCHHPGDIRVLRRTDVPQLRQLKDCIVFPSKGPRPHPDECTGGDLDGDMFVVIWDERLIPSRTQEPMAFD
34. PhysRDR      LDEVDVAVHRSPCHHPGDIRVLRRADVPQLRQLKDCIVFPCQGPRPHPDECTGGDLDGDMFVVIWDKRLIPSRTQEPMAFD
35. PytuRDR      LDQIDVAIHRSPCHHPGDVRVVRRSDVPQLRFLKDCIVFPCDGPRPHPDECTGGDLDGDVFTVIWDERLIPQKENAPMEFQ
36. AlblRDR      LDQVDVVVHRNPCHHPGDIRVLRRADVPQLRCLVDVIVFPVSGPRPHPDECTA---DSEKRKCYRRNGILRRKKKNPENVE
37. PartRDR1     YITGEIIVVRNPCLHPGDIRKVRALSENEIAEYYNCIVFPCKGN-SIPADCGGGDLDGDIYFVSWDPKVIPKTTEYPMNYD
38. PartRDR2     NIQGEVLVTRNPCLHPGDIKKLNAISTQEISQLINCLVFPACGN-SLPCQIAGGDLDGDLYFICWDNRLLPPVQAKSMVYD
39. TettRDR      QVEGDVIVVKNPCLHPGDIRKLKAVTLETIKKLRNILIFPQKGKEPITSQIAGSDLDGDCYFICWDQRFIPLEQKEPFNYD
40. NaegRDR1a    TLSGTVAITRSPCLHPGDIRKFKAVTDPKLSHLRDVIVFSQKGNRPAANKMSGGDYDGDFFQVFWDPLLVNFEEYPPMNYE
41. NaegRDR1b    IVSGNVFVTRNPCLHPGDVRKFKAVS-DVLKHLKDVIVFSQKSKQSQASKMSGGDLDGDLFMVCWDPELLNFTEHKPMKHT
42. HydmRDR1     IIIGDALVYRNPCLHPGDQRVVCCVDEPSLRYLVNVVVFPVMNECSFAASCSGGDLDGDQFAIIWDTKLIPPRHLPRFDYS
43. HydmRDR3     IIIGDALVYRNPCLHPGDQRVVCCVDKPSLRYLVNVVVFPVMNECSFAASCSGGDLDGDQFAIIWDTKLIPPRHLPRFDYS
44. HydmRDR2     IIIGDALIYRNPCLHPGDQRVVCCVDKPSLHYLVNVVVFPVINELSLAASCSGGDLDGDQFAIIWDTKLIPPRHLPRFDYS
45. TrahRDR      VVVGNAIVAKNPCLHPGDIRKVHCVYKKELLHYKNVLVFSRKGTRPTFNMCSGSDLDGDLYFISWDKRLIPRNEHFPQSYQ
46. VavcRDR      VVVGGAIVAKNPCLHPGDIRKVQCVYKKELMHYKNVLVFSKKGRRPVFNMCSGSDLDGDLYFVSWDARLIPPIEHFPQNYQ
47. VitcRDR      VPNCYCIVAKNPVMHPGDIRVVKCVDAPKLHYLKNVIVFSKKGSRPIFNQCSGSDLDGDIFLVSWCKALIPKATFKPYNYI
48. CopcRDR1     YIQGTVSISRSPVAHRGDVQRVYAIGEP-----------PAVGDRSLASCLGGGDVDGDMFSVITCDALLPSETFEPSAYE
49. CopcRDR5     WLEGDCVVTRNPVVRPGDIQKVRAIGKPKCRDLKNVVVFSSQGRRSLPSCLAGGDLDGDKYAVIQYEGLLTEREADPDEAK
50. CopcRDR2     YLEGPVLISRSPTIHPGDVQLVQAIGHPPFEPLANTVVFSVLGDRPLPSCLGGGDLDGDIYNLIPLEGFTPV---RPSQYA
51. CopcRDR3     YLEGDILISRSPCIHPGDVRITRAIGPPPFEPLANTVVFNVLDDRPLASCLGGGDLDGDTYNLLPLSGFRPTRIPEPSQYA
52. NeucRDR1     PTAGPVVVTRSPALHPGDIQIAHNAI-PPLRELKNCIVFSQNGERDLPSQLSGGDLDGDTFNVIWDQSIVATRTFAAADYP
53. EctsRDR1                       CRSVN-------HYTANRASIMTNADRPEPSKMSGGDLDGDLYSVVWDQDLLPPEREPAMGYE
54. EctsRDR2                       CRSVN-------HYTANRASIMTNADRQE---MSGGDLDGDLYSVVWDQDLLPPEREPAMGYE
55. MuccRDR2     VIEGPVVIYRDSSCFPGDVRVMAAINYAELRHYTNVLIFSSIDRCDLPSLCSNDDPDEDNVNVIWDTRLLPVCNVATRSYQ
56. EmihRDR2     VITGRAFFVRNPCLHAGDIRLVDLVELPELAELLN------AGERPLSDQSAGGDLDGDKYLVCWDADIAAARQE
57. DicdRDR1     VIIGRVAVLKNPCHHPGDVRFLKAIDHPPLNGKRNVLFFSTNGMVPNFKQCSGSDLDGDRYFVIYDQTLIRETHE-PYLGE
58. DicdRDR2a    VIEGLVMVIKNPCTHPGDVRYLKAVDNIRLRHLRNVLVFSTKGDVPNFKEISGSDLDGDRYFFCYDKSLIGNRSESETAYL
59. CopcRDR4     PIHGDVLVVRNPCLHPGTV----------LSHLVDCLVFASAGSKPAPSLSSGGDLDGDEFFVCWDQDLVPPRVSEPYDYP
60. NemvRDR3a    QVATEVMVVRNPCYYPGDIRVLKVKNLPQYAYLNDCIVFPTKGSRPHADESSGGDLDGDEFFVSWDQDLIPCWRLPPFDYS
61. NemvRDR3b    QAANKVAVIRNPCYHPGDVRVLKVRNKPELNHLRDCIVFPVRGRRPHALECSGADMDGDKFFVTWDNNLIPRWRVAPYEYG
62. NaegRDR2a    VIENTVIVTKEPCLHRGDLRKLEAVNIPELNFLYDVIVFPTKGSRPITNMIAGSDLDGDQYFACWDNELVESLV-DPGSFD
63. NaegRDR2b    VLQKEIVLTKEPCLHRGDLRKIKAVYYPKLSHLCDVIVFSSKGKRPVPNMISGSDLDGDQFFVCWDDLIVDNVK-EPGLFD
64. NaegRDR6     TLEGTIAITKEPCLHRGDLRLLKAKKIESLEYLSEVIVFPIKGKRPIPNMITGSDLDGDKYLVIWDEPLVSSFVQEPGNFD
65. NaegRDR3     EDGTQVFICRNPCLHPSDIQTFTCRDNSKYRPSNAAIIFPTQG----YTSIKICDFDGDRVFVCWDSRLIPDQVSSPLTHD
66. NaegRDR7     PGGKKVIVFRNPGLHPRDVRIFETVSIPDYGHEVDVIIFAADCQHNIPGELGNFHLDGDRFFTSWNTEIIPQAQY
67. EntdRDR      IPSSRLLVVKFPATHPGDFVLLNVVDYERLTYLRDVIVFSSKGNRPDPNKLSGGDLDGDEYLIIWNPLFLPKNSHPPAIYD
68. EnthRDR      IPSSRLLVVKFPATHPGDFVLLNVVDFERLTYLRDVIVFSSKGNRPDPNKLSGGDLDGDEYLIIWNPLFLPKHSHAPATYD
69. EdhaRDR              VEKDCIKDRKSRKIK-------SDTKNQCFSCTK--------------CGKPFFLSKSC
70. CopcRDR6     IITGDVLITRYPCKLPTDIQKASKVDHPGLHDLVGVIILSTKGTRRAADFIGGGDYDGDKAMIIWHPPMVEAFTPKGHNVP
71. MuccRDR3     QILGDVIVTRNPCALSSDVQKVKAVDCTALRMYSDLAIFSIKGETSLASQLSGGDYDGDIVFCCWDERIVEPFVSTPVPPP
72. MaggRDR      LNDQEVLVARSPAHYVSDIQKVKAVFRPELKDLKDVIVFSRNGNVALADRLSGGDYDGDIAWVCWDSKIVDNFQN-PDSPD
73. NeucRDR6     LSDCDVLVARSPAHFPSDIQRVRAVFKPELHSLKDVIIFSTKGDVPLAKKLSGGDYDGDMAWVCWDPEIVDGFVN-PLEPD
74. GialRDR      CVEGRVVVAKNPCFYRGDVRIYHAVNIPVLGMYRGVICFPKYYGRPMTDCLSGSDLDGDIYWVSWDASLLGTIQREPASFQ
75. AratRDR3a    QISGDVLVYRNPGLHFGDIHVLKATYVKALGNAKFAVFFPQKGPRSLGDEIAGGDFDGDMYFISRNPKLLEHFKPGRKPSE
76. AratRDR3b      SQDVLVYRNPGLHFGDIHILKATYVKSLGNSKYGVFFPQKGPRSLGDEIAGGDFDGDMYFISRNPKLLEHYKPGRQPSE
77. AratRDR3c    QISGEVLVYRNPGLHFGDIHILKATYVKALGNSKFAVFFPQKGPRSLGDEIAGGDFDGDMYFISRNPELLENFKPGRAPSQ
78. OrysRDR3a    QFSGDVLVYKHPGLHFGDIHVLKATYIRDLGYAKYAILFPISGPRSLADEMANSDFDGDIYWVSKNPKLLEHFKP-KKPQD
79. OrysRDR3b    QVSGDVLVYKYPGLHPGDIHVLKATYSSDIGNSKHAILFPTTGQRSLADEMANSDFDGDIYWVSLNPKLLEHFKP-KGPED
80. EmihRDR1     AVLGRCLLYKSPGIHLGDVRAVRAVA-PHLAFAPDGLIVSCQGERSVLDMIAGSDADGDCFTVLFNERLLSLEALRPQPSP
81. NeocRDR      VIQGVVAVCRNPCLFPGDIQLCFAVAL-SLLACRDLIVFPSKGSRDVPNMLSGGDLDGDKFWVTWDPDIVEPLVAPAKAPG
82. ToxgRDR      LIQGVVAVCRNPCLLPGDIQLCFAVGL-GLLACRDVIVFPSARSRDMPNMLSGGDLDGDKFWVVWDPDLLAPLVAPTRSEA
83. PhatRDR      FQENLLFVTRSPCIFPSDGRLLPLVT--KPSLPFGALIFADPGMKPLPAHIANGDLDGDLYFVCWDSEILRNVRADPIVEE
84. ThapRDR1     FGVDQIFVSRSPATEVSDGRVVELVNT-KPSLPFGAIMFGNKGYCPLPECIANGDLDGDWYFVFWSEALLQHIHAIPFIDN
85. ThapRDR2     L--NEVFVSRSPCLTPDDAKLLSVVGS-QPSYRFGTIIFGTNNQTPMPNLIADGDLDGDRYFVLWEPTILDHLQDLTL
86. ThapRDR3     LDQNYVFVTRSPSLAPTDAKLLSVLTS-RPNYKFGTIVFGSNNQTPMPCLIGDGDLDGDDYLVFWESGILSHLLHLTLPET
87. ThapRDR4     RLPSKIFITRSPCTEVSHGRVVEAKELPQFEYSFGSVVFALE-ENPLPMKLADGDLDGDWYFICWDDKILSEVEE
88. NaegRDR4     LELDYVLVYRNPLGYEGDIQKLKIKKNPFLKSRRNSITFAMKGTRNPIGFMQGGDFDGDRYNIIFHPKIVKYDESLNLDYN
89. NaegRDR5     AKIDKVLVYRNPLAYEGDIQCLKLVKHPFLQSRRDVIVFGHNKVPEPLCYMAGGDLDGDLYEIIFDPEIVKLNLKPKLNYN
90. VolcRDR      TGEREVVIYRFPGLHPRDLRKFRSAPPPELPPLTTLLFFSTAGSRAPASGLSGGDYDGDHYTIIWDERVVSQFEEYPPDEE
```

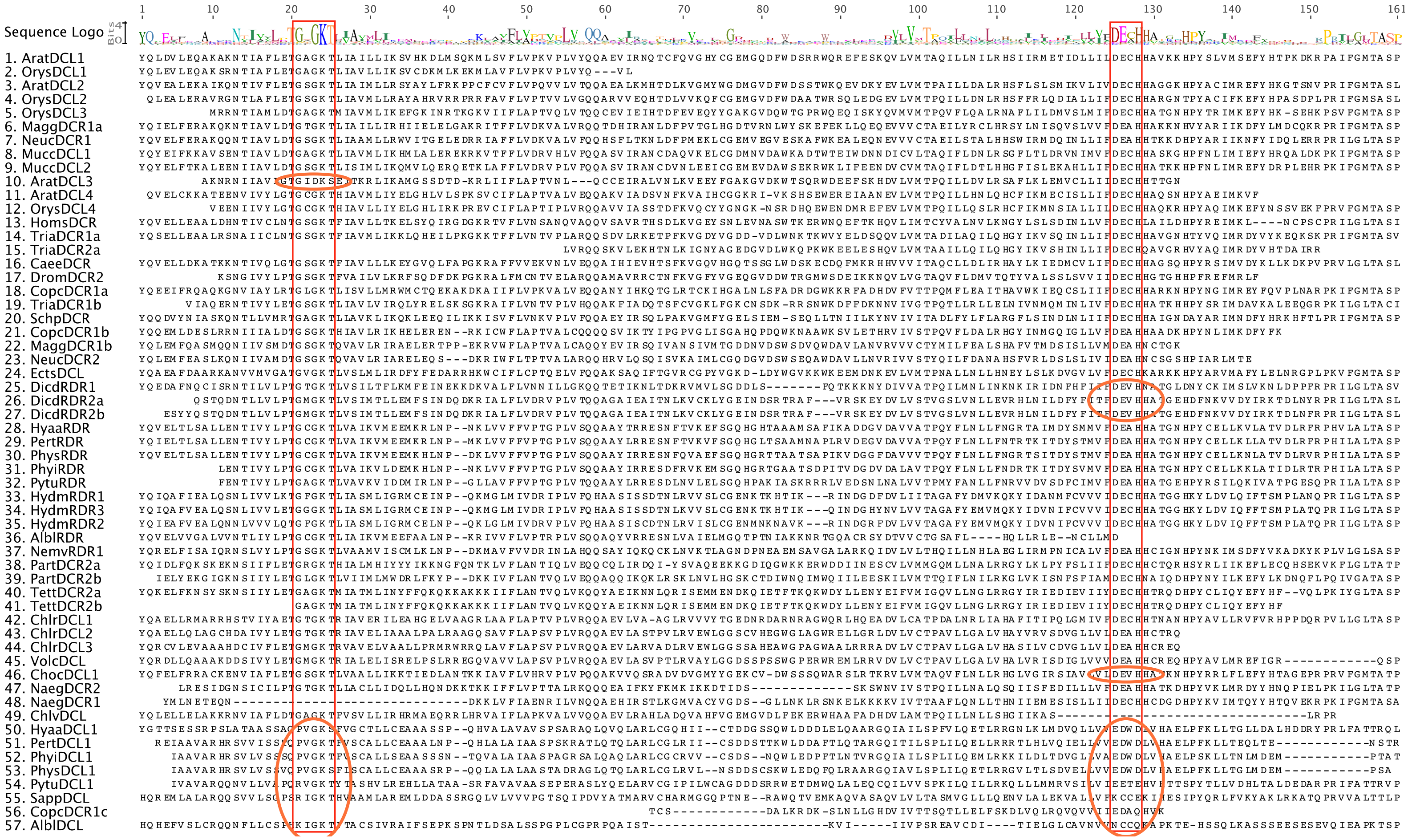

Supplementary Figure 8. Catalytic residues for DEAD-box domain. Alignment of DEAD-box domains from phylogenetic analysis. Red boxes indicate the locus with catalytic activity, which shows strong conservation. Orange ovals indicate deviation from the consensus. Species abbreviations are defined in Supplementary Table 2A.

	ATP-binding residues		Mg^{2+}-binding residues	
	DCL1	RDR	DCL1	RDR
Consensus	GxGKT	GxGKT	DE(C/A)H	DE(C/A)H
P. infestans	PVGKT	GCGKT	EDWD	DEAH
P. sojae	PVGKS	GCGKT	EDWD	DEAH
H. arabidopsidis	PVGKS	GCGKT	EDWD	DEAH
P. tabacina	PVGKT	GCGKT	EDWD	DEAH
P. ultimum	RVGKT	GAGKT	EETE	DEAH
A. laibachii	KIGKT	GCGKT	NCCQ	ERSH
S. parasitica	RIGKT	--	KCCE	--
E. siliculosus	GVGKT	--	DECH	--

Supplementary Figure 9. Conservation of key residues in the DEAD-box helicase domain. Amino acid sequences of the Oomycete DCL1 and RDR homologs at the ATP-binding and Mg2+-binding sites.

Supplementary Table 1. Primers used for cloning

Forward Primer Name	Forward Primer Sequence	Reverse Primer Name	Reverse Primer Sequence	Target Gene	Gene position cloned
5′RACE forward	CGACTGGAGCACGAGGACACTGA	DCL1-5d	AAGGACGCCGACGACAGCACCGGCAGCA	PsDCL1	TSS – 678
5′RACE nested forward	GGACACTGACATGGACTGAAGGAGTA	DCL1-5b	GCGGGTTCGTCACCGGGCTCCAGTCCAT	PsDCL1	TSS – 623
5′RACE nested forward	GGACACTGACATGGACTGAAGGAGTA	DCL1-5c	TCAGCAGCGGGTTCGTCACCGGGCTCCA	PsDCL1	TSS – 629
DCL1-F	ATGGAGGCCGCGCTGTGG	DCL1-R	GTCTTCCTCATCACTCGAGAACGCC	PsDCL1	ATG – term
DCL1-2	GACACGCTGCGGATCTTCG	DCL1-mid1	ATCGTCGTGGACCATTCTTC	PsDCL1	551 – 2796
DCL1-5	CACGGGTGCGATTTTGAACG	DCL1-endB	CTCATCACCGCCGTTGTCC	PsDCL1	2050 – 5038
DCL1-3b	GCAGAAGGCGAGGGGCAAGAGCGCGTCA	3′RACE reverse	GCTGTCAACGATACGCTACGTAACG	PsDCL1	4807 – term
DCL1-3a	GGCGAGGGGCAAGAGCGCGTCAAGTGGA	3′RACE nested reverse	CGCTACGTAACGGCATGACAGTG	PsDCL1	4813 – term
5′RACE forward	CGACTGGAGCACGAGGACACTGA	DCL2-5b	GCGCGGGTTCATCTCGGCCTCCCATTGC	PsDCL2	TSS – 410
5′RACE nested forward	GGACACTGACATGGACTGAAGGAGTA	DCL2-5a	CATGGGCGGTGCTTGTGGCGGGTTGGCT	PsDCL2	TSS – 195
DCL2-F	ATGGACGAAGTGTCCGTTGGGCTC	DCL2-R	GTCTTCGTGCAGTTTGACGCCGTAG	PsDCL2	ATG – term
DCL2-front	GAGATGGAGCACGAGAACG	DCL2-end	GGCATCCTCCTCTGAATCC	PsDCL2	67 – 2815
DCL2-3b	GCCGTTTTGCTGCGCGTTCTGGCGTGGA	3′RACE reverse	GCTGTCAACGATACGCTACGTAACG	PsDCL2	2184 – term
DCL2-3a	CGGCAGTGGATGAACCCGCGCAAGGTGT	3′RACE nested reverse	CGCTACGTAACGGCATGACAGTG	PsDCL2	2657 – term
5′RACE forward	CGACTGGAGCACGAGGACACTGA	RDR-5c	GGGCGTCACCACCACCGCATCAAAGCCT	PsRDR	TSS – 1001
5′RACE nested forward	GGACACTGACATGGACTGAAGGAGTA	RDR-5a	CGTCGCCGCTGTAGTTCGCCCATGCTGT	PsRDR	TSS – 950
5′RACE nested forward	GGACACTGACATGGACTGAAGGAGTA	RDR-5b	CGCTCGTCGCCGCTGTAGTTCGCCCAT	PsRDR	TSS – 954
RDR-front	CGAGCGACCTGGCGTGG	RDR-premid	AATCCGTCCCTGTCGAAAC	PsRDR	73 – 2051
RDR-3	CTGGAAACCACCCTTACTGC	RDR-midc	TTAATCCCAAGGTCGCTCAG	PsRDR	1087 – 4135
RDR-5	CGTACCTGAATGCTCACTGG	RDR-midc	TTAATCCCAAGGTCGCTCAG	PsRDR	2093 – 4135
RDR-6	TGTCGTTCAATCTGCTGTCC	RDR-midc	TTAATCCCAAGGTCGCTCAG	PsRDR	2581 – 4135
RDR-7	TGGTGTTGAAGTCCGTGAAA	RDR-postmid1	ATCAAGCCGACACATTTCCC	PsRDR	3059 – 6064
RDR-10	AATGCACTGGAGGGGATCTA	RDR-postmid1	ATCAAGCCGACACATTTCCC	PsRDR	4651 – 6064
RDR-12	GAAGAACTGCTGGCTGATTTG	RDR-DN50	CTCCCGGTCTGATGCACTTA	PsRDR	5652 – 8209
RDR-3b	TGAAGACAGCGGTGGCGGTGGCGGTGGT	3′RACE reverse	GCTGTCAACGATACGCTACGTAACG	PsRDR	7325 – term
RDR-3a	AGACAGCGGTGGCGGTGGCGGTGGTTCA	3′RACE nested reverse	CGCTACGTAACGGCATGACAGTG	PsRDR	7328 – term

Supplementary Table 2A. Species included in Dicer phylogenetic analyses

Supergroup	Kingdom	Phylum/subgroup	Class/subgroup	Species Name
Archaeplastida	Plantae	Angiosperms	Monocots	Oryza sativa
			Rosids	Arabidopsis thaliana
		Chlorophyta	Chlorophyceae	Chlamydomonas reinhardtii
				Volvox carteri
		Rhodophyta	Florideophyceae	Chondrus crispus
Chromalveolata	Alveolata	Ciliophora	Ciliatea	Paramecium tetraurelia
			Oligohymenophorea	Tetrahymena thermophila
	Stramenopile	Heterokontophyta	Bacillariophyceae	Phaeodactylum tricornutum
			Coscinodiscophyceae	Thalassiosira pseudonana
			Oomycetes	Phytophthora infestans
				Phytophthora sojae
				Hyaloperonospora arabidopsidis
				Peronospora tabacina
				Pythium ultimum
				Albugo laibachii
				Saprolegnia parasitica
			Phaeophyceae	Ectocarpus siliculosus
Excavata	Discicristates	Percolozoa	Heterolobosea	Naegleria gruberi
	Fornicata	Metamonada	Parabasalia	Trichomonas vaginalis
Unikonts	Amoebozoa	Mycetozoa	Dictyostelia	Dictyostelium discoideum
	Animalia	Arthropoda	Insecta	Drosophila melanogaster
		Chordata	Mammalia	Homo sapiens
		Cnidaria	Anthozoa	Nematostella vectensis
		Nematoda	Secernentea	Caenorhabditis elegans
		Placozoa	Tricoplacia	Trichoplax adhaerens
	Fungi	Ascomycota	Ascomycetes	Neurospora crassa
			Saccharomycetes	*Saccharomyces castellii
			Schizosaccharomycetes	Schizosaccharomyces pombe
			Sordariomycetes	Magnaporthe grisea
		Basidiomycota	Agaricomycetes	Coprinopsis cinerea
		Microsporidia	Apansporoblastina	Nosema bombycis
			Microsporidia incertae sedis	Edhazardia aedis
			Pansporoblastina	Trachipleistophora hominis
		Zygomycota	Zygomycetes	Mucor circinelloides
Bacterial outgroups used for RNaseIII domain anaylsis				
Bacteria	Eubacteria	Proteobacteria	Alphaproteobacteria	*Agrobacterium tumefaciens
			Gammaproteobacteria	*Escherichia coli
				*Salmonella enterica
				*Pseudomonas aeruginosa

* outgroups for RNaseIII domain analysis only; genes only had one predicted RNaseIII domain

Supplementary Table 2B. Species included in RDR phylogenetic analysis

Supergroup	Kingdom	Phylum/subgroup	Class/subgroup	Species Name	#RDR	# with DEAD
Archaeplastida	Plantae	Angiosperms	Monocots	Oryza sativa	5	
			Rosids	Arabidopsis thaliana	6	
		Chlorophyta	Chlorophyceae	Coccomyxa subellipsoidea	1	
				Volvox carteri	1	
Chromalveolata	Alveolata	Apicomplexa	Conoidasida	Neospora caninum	1	
				Toxoplasma gondii	1	
		Ciliophora	Ciliatea	Paramecium tetraurelia	2	
			Oligohymenophorea	Tetrahymena thermophila	1	
	Hacrobia	Haptophyta	Prymnesiophyceae	Emiliania huxleyi	2	
	Stramenopile	Heterokontophyta	Bacillariophyceae	Phaeodactylum tricornutum	1	
			Coscinodiscophyceae	Thalassiosira pseudonana	4	
			Oomycetes	Phytophthora infestans	1	1
				Phytophthora sojae	1	1
				Hyaloperonospora arabidopsidis	1	1
				Peronospora tabacina	1	1
				Pythium ultimum	1	1
				Albugo laibachii	1	1
				Saprolegnia parasitica	5	
				Saprolegnia declina	4	
			Phaeophyceae	Ectocarpus siliculosus	2	
Excavata	Discicristates	Percolozoa	Heterolobosea	Naegleria gruberi	10	
Excavata	Fornicata	Metamonada	Eopharyngia	Giardia lamblia	1	
Unikonts	Amoebozoa	Amoebozoa	Archamoebae	Entamoeba dispar	1	
				Entamoeba histolytica	1	
		Mycetozoa	Dictyostelia	Dictyostelium discoideum	3	3
	Animalia	Arthropoda	Arachnida	Ixodes scapularis	4	
		Cnidaria	Anthozoa	Nematostella vectensis	4	1
			Hydrozoa	Hydra magnipapillata	3	3
		Nematoda	Secernentea	Caenorhabditis elegans	4	
	Fungi	Ascomycota	Ascomycetes	Neurospora crassa	3	
			Schizosaccharomycetes	Schizosaccharomyces pombe	1	
		Basidiomycota	Agaricomycetes	Coprinopsis cinerea	6	
		Microsporidia	Apansporoblastina	Vittaforma corneae	1	
			Microsporidia incertae sedis	Edhazardia aedis	1	
			Pansporoblastina	Trachipleistophora hominis	1	
				Vavraia culicis	1	
		Zygomycota	Zygomycetes	Mucor circinelloides	3	
